# Impact of university re-opening on total community COVID-19 burden

**DOI:** 10.1371/journal.pone.0255782

**Published:** 2021-08-12

**Authors:** Lauren E. Cipriano, Wael M. R. Haddara, Gregory S. Zaric, Eva A. Enns

**Affiliations:** 1 Ivey Business School, Western University, London, Canada; 2 Department of Epidemiology and Biostatistics, Schulich School of Medicine and Dentistry, Western University, London, Canada; 3 Department of Medicine, Schulich School of Medicine & Dentistry, Western University, London, Canada; 4 Division of Critical Care, London Health Sciences Centre, London, Canada; 5 Division of Health Policy and Management, University of Minnesota School of Public Health, Minneapolis, MN, United States of America; Columbia University, UNITED STATES

## Abstract

**Background:**

University students have higher average number of contacts than the general population. Students returning to university campuses may exacerbate COVID-19 dynamics in the surrounding community.

**Methods:**

We developed a dynamic transmission model of COVID-19 in a mid-sized city currently experiencing a low infection rate. We evaluated the impact of 20,000 university students arriving on September 1 in terms of cumulative COVID-19 infections, time to peak infections, and the timing and peak level of critical care occupancy. We also considered how these impacts might be mitigated through screening interventions targeted to students.

**Results:**

If arriving students reduce their contacts by 40% compared to pre-COVID levels, the total number of infections in the community increases by 115% (from 3,515 to 7,551), with 70% of the incremental infections occurring in the general population, and an incremental 19 COVID-19 deaths. Screening students every 5 days reduces the number of infections attributable to the student population by 42% and the total COVID-19 deaths by 8. One-time mass screening of students prevents fewer infections than 5-day screening, but is more efficient, requiring 196 tests needed to avert one infection instead of 237.

**Interpretation:**

University students are highly inter-connected with the surrounding off-campus community. Screening targeted at this population provides significant public health benefits to the community through averted infections, critical care admissions, and COVID-19 deaths.

## Introduction

In response to the novel coronavirus pandemic, universities have had to make decisions about how to conduct their academic year. Some universities opted to operate fully online for the fall term [1]; others are attempting to bring students back to campus with varying levels of mitigation, including mandated face-coverings, limiting large gatherings, frequent COVID-19 screening, reduced dormitory occupancy, and accommodations for isolating and quarantining students [2].

University students are themselves generally at lower risk for severe COVID-19 outcomes. However, university students live, work, and socialize both on and off campus, and so there is significant potential for COVID-19 outbreaks in the university population to spill over into the community and exacerbate existing community burden [3]. Thus, mitigation measures adopted by university leaders for the university community may have substantial public health implications for the surrounding community.

Several studies have modeled COVID-19 transmission dynamics, tailored to reflect a university context, to evaluate testing and contact tracing strategies [4–9]. Largely focusing on different frequencies of screening students for coronavirus infection, these studies concluded that frequent testing would be needed to contain COVID-19 outbreaks on campus [4–8]. However, these studies focused entirely on outcomes occurring in the university population. While some studies did include infections among students arising from off-campus community contact, none considered the impact of university management decisions and university student contact patterns on the infection risk in the broader community.

To address this gap, we developed a dynamic model of COVID-19 transmission in a representative mid-sized city with a relatively large destination college campus. We assumed a city initially experiencing a low level of COVID-19 activity going into the fall prior to the on-campus arrival of the university student population. Under different scenarios of community physical distancing effort and routine testing in students, we used the model to estimate the incremental COVID-19 burden attributable to the arrival of the students.

## Methods

**Fig 1:**
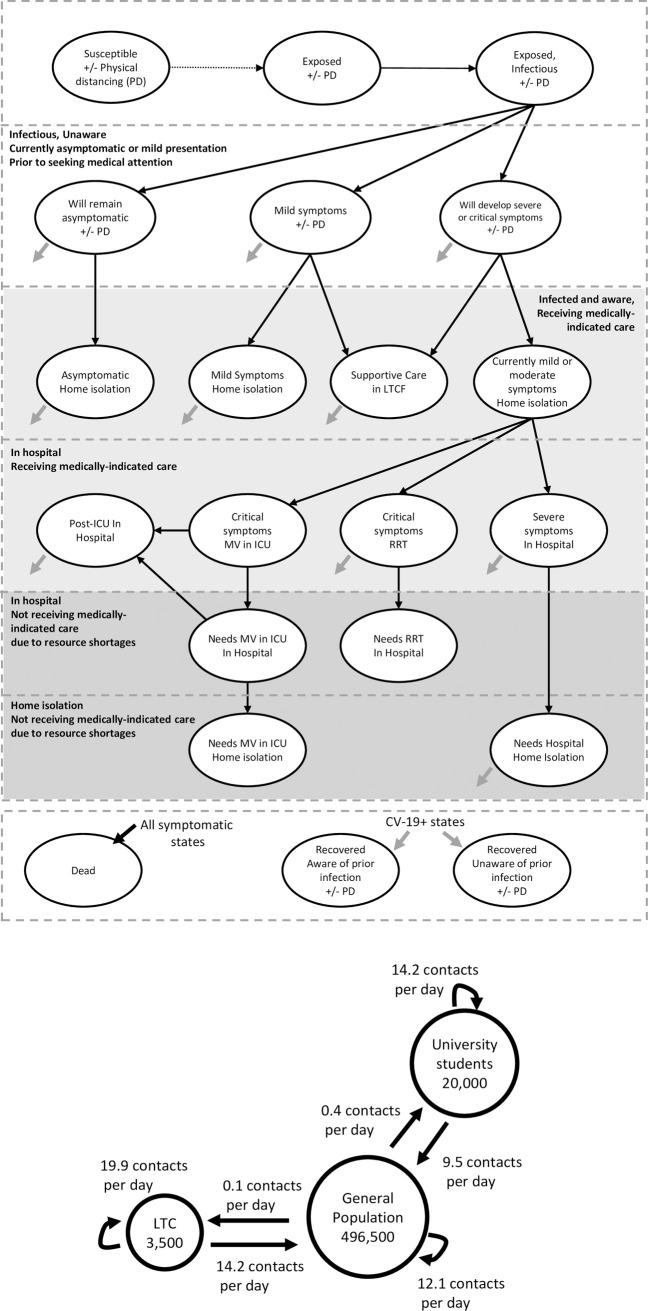
Model schematics of (A) COVID-19 health states and (B) close contact interactions between population subgroups in the pre-COVID era. In the model, susceptible individuals may become infected through interaction with infected individuals who may or may not be aware of their infection status. Infection has a pre-symptomatic phase in which an infected individual can transmit the infection to others. Individuals may become aware of their infection status through symptom-based surveillance, contact tracing, or routine testing of asymptomatic and mildly symptomatic individuals. Individuals aware of their infection status with mild or moderate symptoms isolate at home to reduce disease transmission. Some patients develop severe symptoms requiring hospitalization or critical symptoms requiring mechanical ventilation (MV) in an intensive care unit or renal replacement therapy (RRT). Patients receive medically indicated care unless resource demand exceeds capacity. When hospital capacity for a medically indicated resource has been reached, patients receive the next-best available care. The average number of contacts per day for the general population was estimated using an extrapolation of the 2008 POLYMOD data to a Canadian setting [10]. For university students, average contacts per day was estimated based on surveys in this population [11]. Average contacts per day for long-term care residents were estimated using a study in which residents and staff were equipped with RFID tags [12, 13].

We developed a dynamic compartmental model (**Fig 1**) to simulate infection dynamics and health resource use of a representative mid-sized city with a population of 500,000 going into fall after experiencing low rates of COVID-19 infections in the summer. We divided the population into three interacting sub-populations with different numbers and patterns of contacts: long-term care (LTC) residents, university students, and the general population. We evaluated COVID-19 health outcomes in the city over a period of 4.5 months (August 15 to December 31), beginning two weeks prior to the arrival of 20,000 university students on September 1. Institutional ethics review was not required for this modeling study as human subjects were not involved.

We estimated model parameters, including the duration of time spent in each health state, the infectiousness of COVID-19, demand for hospital resources and disease mortality conditional on disease severity, and the effectiveness of COVID-19 prevention strategies using the peer-reviewed literature, pre-published reports, and expert opinion (**Table 1**). We calibrated uncertain model inputs to the observed hospitalization and mortality outcomes in London, Canada, a mid-size city with a large university population, between March 1 to August 15. Full details are presented in the **Supplemental Methods in S1 File**.

**Table 1:** Base case parameters and sources.

Parameter	Mean and 95% CI	Reference
**Contact structure (contacts per day)**		
*General population*	12.60	[10]
General population	12.12	Calculated [10]
University students	0.38	Calculated [10]
Long-term care residents	0.10	Calculated
*University students*	23.70	
General population (includes faculty, staff, and graduate students)	9.48 (5.0, 15.0)	Calculated [11, 14]
University students	14.22 (10.0, 28.4)	Calculated [11, 14]
Long-term care residents	0	Assumed
*Long-term care residents*	34.1	[13, 15]
General population (includes LTC staff)	14.2 (11.4, 17.0)	[13, 15]
University students	0	Assumed
Long-term care residents	19.9 (11.3, 28.5)	[13]
**Infectiousness and COVID-19 prevention behaviours**		
R0: Average number of new infections per infection	3.0 (2.85, 3.3)	Empirically estimated ^a^
Reduction in contacts when aware of infected status and in-home isolation	90% (80%, 95%)	Assumed
Reduction in contacts when in hospital	100%	Assumed
Effectiveness of mask wearing, reduction in transmission during a close contact between a susceptible and an infected person	40%	[16]
*General population*		
Initial proportion who are ‘high-intensity physical distancers’	40%	[17]
*High-intensity physical distancer*		
Reduction in contacts	75%	[17]
Mask wearing	86%	[17]
*Low-intensity physical distancers / Unable to reduce contacts*		
Reduction in contacts	30%	Calculated ^g^
Mask wearing	38%	[17]
*University students*		
Initial reduction in contacts	40%	Calculated ^h^
Mask wearing	57%	[17]
*Response to COVID-19 community outcomes*		
*General population increase participation in high-intensity physical distancing*		
COVID-19 patients in critical care exceeds 15	0.5% per day	Assumed
COVID-19 deaths in the past 10 days exceeds 10	1% per day	Assumed
Maximum level of participation in high-intensity physical distancing	80%	Assumed
*University students increase reduction in contacts*		
COVID-19 patients in critical care exceeds 15	0.5% per day	Assumed
COVID-19 deaths in the past 10 days exceeds 10	1% per day	Assumed
Maximum level of contact reduction	50%	Assumed
**Time to diagnosis**		
Minimum time from symptom onset to clinical presentation (average days)	2.1 (1, 3)	
*Daily probability of diagnosis by symptom-based surveillance and contact tracing*, *general population and student population*		
Symptomatic cases	15.8%	Calculated ^b^
Asymptomatic cases	4.1%	Calculated ^b^
Sensitivity of nasopharyngeal swab PCR test for COVID-19	72.1%	[18, 19]
**Disease severity distribution**		
*Long-term care residents*		
Asymptomatic	12% (1.2%, 22.6%)	[20, 21]
Symptomatic, cared for in long-term care	76.2%	Calculated
Hospitalized, no critical care resources	11.4% (9%, 14%)	[22]
Critical, requires mechanical ventilation (MV)	0.3% (0, 0.7%)	[22]
Critical, requires renal replacement therapy (RRT)	0.1% (0, 0.2%)	Estimated ^c^
*University students*		
Asymptomatic	31% (18%, 80%)	[23]
Mild or Moderate	67.8%	Calculated
Severe	1.0% (0.5%, 1.5%)	Estimated ^d^
Critical, requires MV	0.18% (0, 0.4%)	[24]
Critical, requires RRT	0.06% (0, 0.1%)	Estimated ^c^
*General population*		
Asymptomatic	31% (26%, 37%)	[23]
Mild or Moderate	60.4%	Calculated
Severe	3.75% (2.0%, 8.0%)	Calibrated ^e^
Critical, requires MV	1.25% (1.0%, 1.8%)	[24]
Critical, requires RRT	0.45% (0.2%, 0.7%)	Estimated ^c^
**Time to health event transition (Mean** ^**f**^ **, days)**		
Average duration of infectiousness	10 (6.3, 16.0)	[25–28]
Incubation period: Exposure → Symptom onset	5.6 (5.1, 6.1)	[29]
Infectiousness prior to symptom onset	2.5 (2.0, 3.0)	[25, 27, 28]
Diagnosis: Symptom onset → First opportunity for diagnosis	2.1 (1.1, 3.1)	[30]
Symptom onset → Progression to severe or critical symptoms	5.8 (4, 8)	[31]
Severe symptoms: In hospital → Recovery or Death	8.3 (6, 12)	[31]
Critical care: MV in ICU → Post-ICU in hospital or Death	15.5 (10, 32)	[32]
Critical care: Post-ICU → Recovery	10.1 (6, 18)	[32]
Critical: RRT → Discharge or Death	25.0 (12, 44)	[32]
Symptomatic in LTC: Symptom onset → Recovery or Death	18.0 (14, 24)	Estimated in calibration
*Clinical improvement in patients receiving lower level of care than is medically indicated*		
Severe symptoms: Home isolation → Recovery	18.0 (14, 24)	Assumed
**Mortality**		
*Long-term care residents*		
Symptomatic, cared for in long-term care	25.5% (21%, 30%)	[22]
Hospitalized, no critical care resources	47.4% (34%, 60%)	[22]
Critical, requires MV	70.8% (66%, 75%)	Based on outcomes in ≥ 70 year olds [32]
Critical, requires RRT	74.9% (67%, 83%)	Based on outcomes in ≥ 70 year olds [32]
*University students*		
Mild or Moderate	0%	
Severe (In hospital)	0.43% (0.1%, 0.7%)	Estimated non-ICU mortality for < 55 year olds [31]
Critical, requires MV	21.5% (17%, 25%)	Based on outcomes in 16 to 39 year olds [32]
Critical, requires RRT	35.9% (26%, 46%)	Based on outcomes in 16 to 39 year olds [32]
*General population*		
Mild or Moderate	0%	Assumed
Severe (In hospital)	14.4% (4%, 33%)	Estimated non-ICU mortality for < 75 year olds [31]
Critical, requires MV	42.9% (41%, 45%)	Based on outcomes in < 70 year olds [32]
Critical, requires RRT	53.4% (50%, 57%)	Based on outcomes in < 70 year olds [32–34]
*Mortality in patients unable to receive medically indicated care*		
Case fatality rate, Severe patient requiring hospitalization, In home isolation	25% (16%, 35%)	Assumed
Daily rate, Patients who need MV or RRT, In hospital	40% (21%, 60%)	Assumed, 2-day life expectancy
Daily rate, Patients who need MV or RRT, In home isolation	60% (41%, 80%)	Assumed, 1-day life expectancy

Mean and 95% confidence interval representing the uncertainty in the mean used in sensitivity analysis.

a. Using exponential regression, we empirically estimated the basic reproduction number, R_0_, the average number of secondary infections produced by one infected individual during the infected individual’s entire infectious period assuming a fully susceptible population, is 3.0 based on Ontario’s reported cases between March 7 to March 22 [35].

b. The observed median time to diagnosis through symptom-based surveillance alone of 4.6 days (95%CI: 4.2, 5.0) and symptom-based surveillance in combination with contact tracing efforts of 2.9 days (95%CI 2.4, 3.4) in Shenzhen, China [36]. From this, we estimated that symptom-based surveillance and contact tracing results in a daily probability of diagnosis of 15.8% and the daily probability of detection from contact tracing of 4.1% in asymptomatic infections.

c. Among critical care patients, we estimate the ratio of patients requiring renal replacement therapy (RRT) to mechanical ventilation (MV) based on the UK Intensive Care National Audit and Research Centre (ICNARC) report describing the care and outcomes of 10,118 critical care COVID-19 patients in the UK. In this report, 7,277 patients required MV and 2,673 required RRT, resulting in a ratio of 0.37 RRT patients per mechanical ventilation patient [32].

d. In Canada, based on 63,800 COVID cases in people who were not residents of long-term care facilities reported between February 23 and June 21, 20.3% of hospitalized patients received critical care [22]; this is also consistent with rates of critical care observed in the UK (22% overall hospitalized patients go to ICU) [31]. Therefore, we estimate the ratio of 3.92 hospitalized without critical care patients per critical care patient.

e. Initially estimated using the same process as is described in footnote d. Adjusted in calibration process to better fit the observed data (see **Supplemental methods in S1 File).**

f. Median and IQR presented in the cited primary work were transformed to Mean (95%CI range) assuming a gamma distribution.

g. Due to reduced density in public spaces and reduced availability of their usual contacts, low-intensity physical distancers also experience an overall contact reduction calculated at each time to be equal to the reduction in overall contacts imposed by the ‘high intensity physical distancers’ [Proportion of the population that are ‘high intensity physical distancers’ × 75% reduction in contacts].

h. Calculated as 32% self-reporting high-intensity physical distancing × 75% reduction in contacts and the remainder having reduced contacts due to the reduced access to their usual contacts and reduced population density in public spaces. So, 32% × 75% + (1–32%) × (32% × 75%) = 40.3%.

### Community behaviour

We subdivided the general population into two groups based on intensity of COVID-19 prevention behaviours as described in a recent poll of Canadians [17]. ‘High-intensity physical distancers’, representing 50% of the general population initially, reduce their average number of contacts by 75% (from 12.6 to 3.2 contacts per day) and 86% of their remaining contacts are protected by a cloth mask. We assumed that the remainder of the population reduce contacts proportional to the overall reduction in contacts imposed by ‘high-intensity physical distancers’ which can be attributed to reduced availability of their usual contacts and reduced density in public spaces. Further, we assume they are using a cloth mask to protect 38% of those contacts. In the base case, we assumed that university students initially reduce their contacts by 40% (from 23.7 to 14.1 contacts per day) and that 57% of contacts were protected using a mask [17]. We assumed cloth masks reduce disease transmission by 40% [16].

#### Responsive physical distancing behaviours

We assumed that the general population and university students respond to COVID-19 outcomes in the community, specifically reduced access to non-COVID health services and COVID-19 mortality, by increasing their protective behaviours, as observed in a US-based study [37]. These changes in behaviour are intended to capture both individual decision-making and policy changes instituted by the city. We relied on locally relevant thresholds to inform triggers for community behaviour change. Based on expert opinion, substantial reductions in access to other health care services would need to occur if 30 critical care beds (about 40% of normal critical care capacity in a city of 500,000 [38]) were occupied by COVID-19 patients. So, if the number of COVID-19 patients in critical care exceeds 15, we assumed that the proportion of the general population who are ‘high-intensity physical distancers’ increases by 0.5% each day and, if the number of COVID-19 deaths in the past 10 days exceeds 10, we assumed that the proportion of the general population who are ‘high-intensity physical distancers’ increases by 1.0% each day, up to a maximum of 80% participation in high-intensity physical distancing. Similarly, we assume students increase physical distancing at the same rate in response to the same triggers, but up to a maximum of a 75% reduction in contacts.

### Diagnosis by symptom-based surveillance, contact tracing, and routine testing

We estimated that symptom-based testing and contact tracing results in a daily probability of diagnosis of 15.8% for symptomatic infections and a daily probability of detection (from contact tracing) of 4.1% for asymptomatic infections [36]. We considered policy alternatives of routine screening for COVID-19 in university students at various screening frequencies and one-time universal screening three weeks after student arrival. We assumed that testing will be performed by PCR analysis of a nasopharyngeal swab with a test sensitivity of 72.1% [18, 19]. For people who are aware of their infection status, we assume a 90% reduction in contacts [39, 40].

## Results

**Fig 2:**
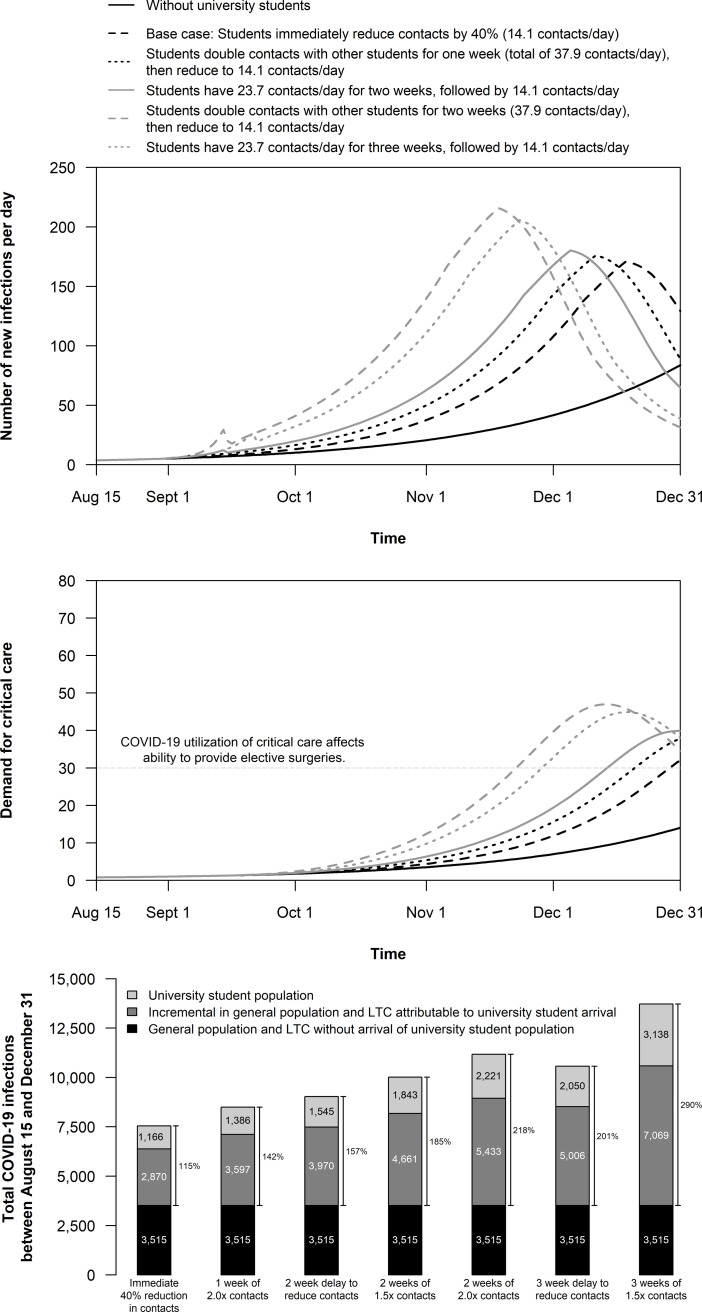
Epidemic outcomes in a city of 500,000 with and without the introduction of 20,000 university students on September 1. Scenarios consider different initial physical distancing behaviours in the university student population. (A) Number of new infections per day; (B) the number of people medically indicated for critical care each day; and, (C) the cumulative number of COVID-19 infections between August 15 and December 31. Numerical results are provided in **Appendix Table 4 in S1 File**.

Under base case assumptions without the introduction of the student population, the simulated city experiences a total of 3,515 infections over 4.5 months. In the base case with student arrival, we conservatively assumed that students would bring no undiagnosed infections of COVID-19 to the community and would immediately engage in physical distancing efforts. Even so, the introduction of students to the community increases the total number of infections by 4,036 infections, an 115% increase (from 3,515 to 7,551) (**Fig 2**). Of the incremental infections, 70% occur in the general population, which in turn increased COVID-19 hospitalizations and deaths and caused COVID-19 critical care demand to exceed 30 beds before the end of term (**Appendix Table 2 in S1 File**).

If, instead, students have twice the pre-COVID era number of contacts with other students for the first two weeks (28.4 contacts with other students, resulting in a total of 37.9 contacts per day) after which they implement a 40% reduction in their contacts (from an average of 23.7 total contacts reduced to 14.1 total contacts per day), then the total number of infections in the community increases by 7,654, leading to an additional 83 COVID-19 deaths (**Appendix Fig 8 and Appendix Table 4 in S1 File**). Short-term increases in the number of student-student contacts increases demand for critical care resources and shortens the time until COVID-19 critical care demand exceeds 30 beds (**Fig 2B**).

### Effectiveness of routine asymptomatic screening targeted at students

**Fig 3:**
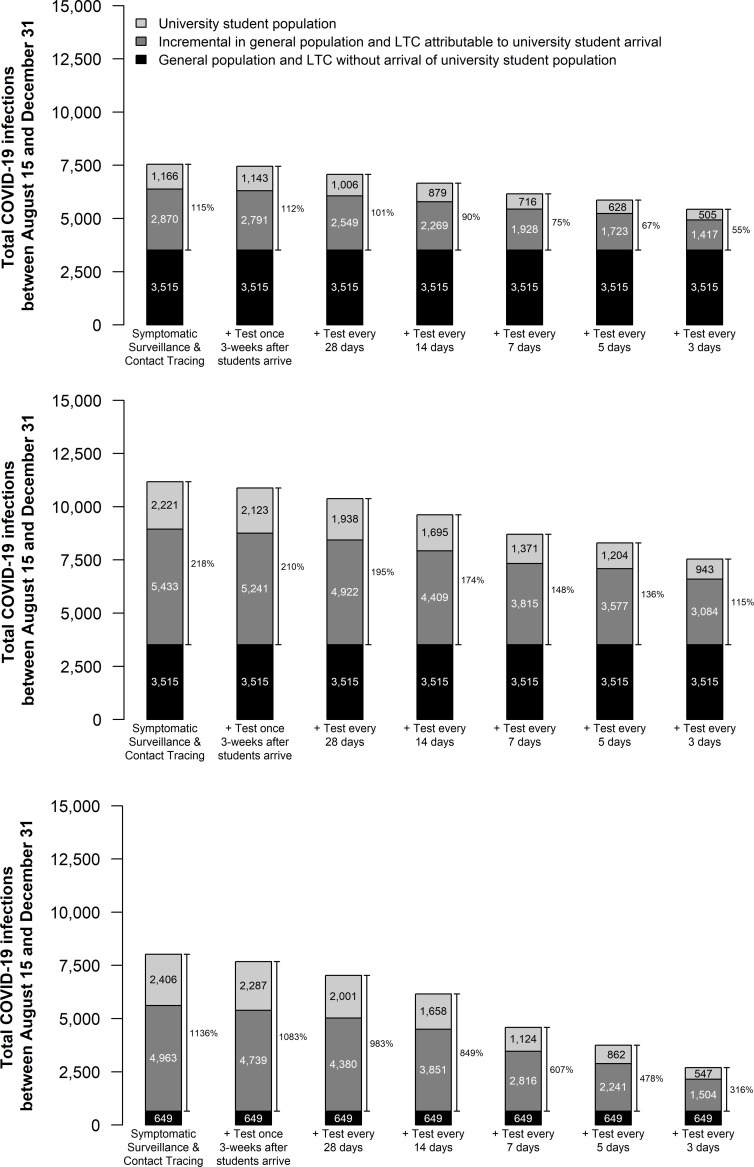
Cumulative number of COVID-19 infections between August 15 and December 31 in a city of 500,000 with and without the introduction of 20,000 university students on September 1. Scenarios in each panel differ in the frequency with which students undergo routine testing for COVID-19. In panel (A), students have an average 40% reduction in contacts compared to normal student social interaction behaviour (average of 23.7 contacts reduced to 14.1 contacts) immediately upon arrival with no short-term increase in contacts; in panel (B), students double their contacts with other students for the first two weeks (28.4 contacts with other students, resulting in a total of 37.9 total contacts per day) and then implement a 40% reduction in their baseline contacts (average of 23.7 total contacts reduced to 14.1 total contacts); in panel (C), students double their contacts with other students for the first two weeks and then implement a 40% reduction in contacts and 50% of the general population is participating in high-intensity physical distancing (compared to 40% in the base case and other scenarios presented in this figure). Other outcomes for these scenarios are reported in **Appendix Table 5 in S1 File**.

Testing students every 28 days results in very little reduction in the number of infections but requires a large number of tests (714 students tested per day) (**Fig 3**). More frequent testing reduces infections further. Across scenarios, routine testing of students every 5 days reduces the total number of infections attributable to the return of university students by 42% (from 4,036 to 2,351 in the base case) (**Appendix Table 5 in S1 File**). This strategy averts substantial numbers of critical care admissions and COVID-19 deaths in the because approximately two-thirds of averted infections are prevented in the general population (**Table 2**). Accounting for the costs of hospitalizations, economic value of deaths averted, and productivity costs of infections averted, a 5-day testing strategy would be cost-effective if testing cost less than $59 per test under the most conservative assumptions of student contact behaviour (**Appendix Table 7 in S1 File**). Higher levels of student contacts increase the cost per test that would be considered cost-effective.

**Table 2:** Infections averted in the general population with 5-day testing and one-time testing of students compared to a policy of no routine asymptomatic testing (symptom-based surveillance and contact tracing only).

			5-day testing compared to no routine testing				One-time testing three weeks after student arrival compared to no routine testing			
Scenario	Without students	With students	Infections averted	% averted in the general pop’n	Critical care adm’n averted	COVID-19 deaths averted	Infections averted	% averted in the general pop’n	Critical care adm’n averted	COVID-19 deaths averted
**31% asymptomatic in students**										
Base case physical distancing (40% reduction in contacts immediately)	3,515	7,551	1,685	68%	19.3	15.0	102	76%	1.3	1.0
Two weeks of 2.0x activity among students, followed by 40% reduction in contacts	3,515	11,169	2,873	64%	31.3	24.3	290	66%	3.2	2.5
Low level of physical distancing (24% reduction in contacts immediately)	3,515	14,263	5,451	57%	52.5	40.8	186	63%	2.0	1.5
Two weeks of 2.0x activity among students, followed by 40% reduction in contacts; 50% of general population engaged in high-intensity physical distancing	649	8,018	4,266	63%	45.6	35.4	343	65%	3.8	2.9
**50% asymptomatic in students**										
Base case physical distancing (40% reduction in contacts immediately)	3,515	7,632	1,768	67%	20.2	15.7	51	69%	0.6	0.5
Two weeks of 2.0x activity among students, followed by 40% reduction in contacts	3,515	11,076	2,789	62%	29.6	23.0	329	66%	3.7	2.9
**80% asymptomatic in students**										
Base case physical distancing (40% reduction in contacts immediately)	3,515	7,794	1,934	67%	22.0	17.1	69	71%	0.8	0.7
Two weeks of 2.0x activity among students, followed by 40% reduction in contacts	3,515	11,514	3,238	64%	35.0	27.2	419	68%	4.9	3.8

Scenarios vary the proportion of infections in the student population that are asymptomatic and timing and level of students contact reductions. We calculate the expected number of critical care admissions averted and COVID-19 deaths averted to be 1.7% and 1.32% of general population infections averted which includes hospitalizations and deaths which may occur after December 31 to all individuals infected prior to December 31. The economic value of testing strategies is presented in **Appendix Table 7 in S1 File**.

Sensitivity analysis revealed that routine testing of university students was more valuable when students have a higher rate of asymptomatic infections (**Table 2**) and in scenarios in which the differences in transmission risk between the university students and the general population were greater. For example, in a scenario in which the city had a high level of engagement in physical distancing, routine screening of the student population averts a larger fraction of infections because in these scenarios the city expects very little COVID-19 transmission without the introduction of the student population (**Fig 3C**). Conversely, in scenarios in which the city is engaged in a low level of physical distancing, and so expects a large number of infections with or without the student population, the difference in risk profile between the city and the university populations decreases, as does the benefits of targeting prevention efforts to the university population.

### Effectiveness of one-time screening targeted at students

Routine testing to identify and isolate asymptomatic infections for the purposes of reducing community transmission risk requires a high volume of tests each day and may strain community testing resources. We also evaluated the benefits of a one-time universal screening event occurring three weeks after the students arrive. Through the isolation of identified cases, one-time testing immediately decreases incident infections in the student population and, indirectly, in the general population (**Appendix Fig 9 in S1 File**). In the case that students double their contacts with other students for a period of two-weeks, this strategy prevents 290 infections, 3.2 critical care admissions, and 2.5 COVID-19 deaths (**Table 2**); however, one-time screening does not significantly impact the timing of peak infections, resource utilization (**Appendix Table 5 in S1 File**). Depending on student behaviours, one-time screening could be cost-effective if the test costs less than $200 per test (**Appendix Table 7 in S1 File**).

## Discussion

We analyze the COVID-19 impacts of re-opening a destination university in a mid-sized city with varying epidemiological contexts. Though the severity of COVID-19 in the fall depends on the level of preventive behaviours in the general population, the arrival of students always worsens COVID-19 outcomes, even under conservative assumptions. This is because university students have nearly double the number of contacts as the general population due to higher levels of shared living situations, service sector employment, and social activity. In the scenarios we considered, this increase in infections was substantial, potentially doubling of the total number of COVID-19 infections in the city over the fall, with more than two-thirds of the incremental infections occurring in the general population. This substantially impacted the number of COVID-19 hospitalizations and deaths in the community and also caused critical care utilization to reach levels that would require reductions in non-COVID health care services.

Previous studies modeling university populations did not account for infections in the broader community [4–8]. However, we have shown that including the city population is critical for decision making, since this population bears the brunt of the incremental morbidity and mortality burden of COVID-19. The number of infections is directly proportional to the total number of contacts in the community. Therefore, cities can also balance the increased risk of returning university students by heightening physical distancing measures. For example, in the base case, the increase in infections due to student arrival could be completely mitigated if the proportion of the general population engaged in high-intensity physical distancing increased by 5.8% (from 40% to 45.8%). This is because having approximately 23,000 (5.8% of 500,000) additional people in the community reduce their contacts by 75% (from 12.6 to 3.15 contacts per day) and wear a partially effective mask with 86% of their remaining contacts balances with the increased contacts introduced by the 20,000 university students with 14 contacts per day and 57% mask wearing. This illustrates the idea of “risk budgets”, where increased risk in one domain of a community necessitates reducing risk in another to keep COVID-19 impacts below desired thresholds [41], and highlights the need for coordination between university decision-makers and the broader community.

Our analysis indicates that routine screening of all students every 5 days averts a substantial number of infections, critical care admissions, and COVID-19 deaths. In the base case, we estimate that testing every 5 days prevents 19.3 critical care admissions and 15.0 deaths and nearly double these values in the scenario in which students double their contacts with other students for two weeks. Using a relatively simplistic economic analysis, we estimate that this testing frequency would be cost-effective if the test could be performed for a cost of $36 to $59 per test. Because the current cost of a nasopharyngeal COVID-19 test is $80 at our center, high-frequency testing may be cost-prohibitive. Alternatively, one-time universal testing of students after an initial burst of social activity among students may be more operationally and economically feasible. This strategy still averts 290 infections, 3.2 critical care admissions, and 2.5 COVID-19 deaths in the scenarios in which students double their contacts with other students for two weeks, corresponding to cost-effective strategy if the test can be provided for less than $123 to $199 per test. Our economic analysis underestimates the benefits of testing by not accounting for savings due to averted critical care admissions and the community economic benefits of delaying social and economic restrictions. Further, a one-time testing strategy may provide high-quality data on the status of the epidemic to inform future decisions.

Compared to other modeling studies of COVID-19 on university campuses, the total number of infections and the number of infections averted by testing estimated in our analysis are modest. This is because, in our analysis, testing is being layered onto a robust and reactive mitigation response in which both university students and the general population are assumed to increase self-protective behaviours in response to high numbers of COVID-19 hospitalizations and deaths. This creates a feedback loop moderating the magnitude of the increase in infections associated with higher levels of contacts. This moderating effect occurs because the community exceeds the COVID-19 outcomes leading to increased protective behaviours sooner. Therefore, the earlier need for these measures means that normal activities will be disrupted sooner and possibly require formal social and economic restrictions to enforce protective behaviours.

An important limitation of our analysis is the assumption that students self-isolate effectively. However, students may not be able to isolate from roommates or refrain from using shared facilities, like bathrooms and kitchens, without dedicated university-organized isolation facilities [42, 43]. Furthermore, adherence to isolation guidance may be low, especially for asymptomatic or mild cases. During the H1N1 influenza pandemic, a survey of symptomatic university students found that only 41% of students followed recommendations to stay home until well [44]. In the base case, we also assume that students are equally responsive in adopting self-protective behaviours as the general population when COVID-19 hospitalizations and deaths reach high levels. The extent and speed with university students respond to COVID-19 in the local community impacts the number of infections experienced by the community and the benefits of routine testing in the student population.

Our analysis is relevant to a number of mid-sized cities in North America with relatively large university and college populations. Because university students have substantially more contacts than the general population, the introduction of university students can substantially increase the number of COVID-19 infections and decreases the time until responsive behaviours are activated. Substantial uncertainty exists in the level of contact reduction that students will choose, or is feasible given their living, transit, and work situations. Public health interventions, such as routine testing, targeted at this population prevents infections in the entire population, improving community health related and unrelated to COVID-19. The importance of targeting prevention efforts to the student population is greatest when there is substantial difference in the contact behaviour between students and the general population. If the general population does not adopt public health measures such as high-intensity physical distancing and mask wearing, the return of students will not be the main driver of community outcomes. However, we consistently find, across scenarios with varying levels of social distancing in the general population, the number of general population infections attributable to the return of the student population is about twice the number that occurs in the student population itself.

## Supporting information

S1 File The supplemental material contains supplemental methods, with supporting Appendix Figs 1 through 4, and supplemental results, with supporting Appendix Figs 5 through 11 and Appendix Tables 1 through 7. (PDF)

S2 File (R)

S1 Table (XLSX)
