# Supplementary material for: Impact of university re-opening on total community COVID-19 burden
Source: PLoS One. 2021 Aug 12;16(8):e0255782. doi: 10.1371/journal.pone.0255782 (PMC8360395; doi:10.1371/journal.pone.0255782)
Supplement: S1 File — (PDF) [file pone.0255782.s001.pdf]

IMPACT OF UNIVERSITY RE-OPENING ON TOTAL COMMUNITY COVID-19 BURDEN

Cipriano LE, Haddara WMR, Zaric GS, Enns EA

July 30, 2021

Supplemental Material

SUPPLEMENTAL METHODS

OVERVIEW

We developed a deterministic dynamic compartmental model of the COVID-19 pandemic that simulates the health outcomes and resource use in a representative mid-sized city with a full-time population of 500,000 plus an additional part-time academic year population of 20,000 university students. We use the model to project how COVD-19 risk and prevention behaviours of the general population, and the student community in particular, affect the incremental COVID-19 burden attributable to the arrival of 20,000 university (post-secondary) students on September 1. Under different scenarios of community physical distancing effort and testing in students, we calculated the number of infected individuals, hospital resource demand, and health outcomes overall and over time. The model was implemented in Microsoft Excel in Microsoft Office 365. Institutional ethics review was not required for this modeling study.

A schematic of the model is presented in **Figure 1**. In the model, susceptible individuals may become infected through interaction with infected individuals who may or may not be aware of their infection status. Infection has a pre-symptomatic phase in which an infected individual can transmit the infection to others [1-3]. Individuals may become aware of their infection status through symptom-based surveillance, contact tracing, or routine testing of asymptomatic and mildly symptomatic individuals. Individuals aware of their infection status with mild or moderate symptoms are assumed to isolate at

home to reduce disease transmission. Some patients develop severe symptoms requiring hospitalization or critical symptoms requiring mechanical ventilation (MV) in an intensive care unit or renal replacement therapy (RRT). Patients receive medically indicated care, unless resource demand exceeds capacity. When hospital capacity for a medically indicated resource has been reached, patients receive the next-best available care.

Interventions such as physical distancing, which reduced the average number of contacts between susceptible and infected people, or mask wearing, which reduce the probability of disease transmission between contacts, each reduce the expected number of infections in the time periods when they are adopted. Further, we assumed that the general population and university students respond to COVID-19 outcomes in the community. In practice, this response may be voluntarily adopted due to public concern over reported increases in COVID-19 cases, hospitalizations, and/or deaths or imposed through policies that re-establish the social and economic restrictions utilized in the earlier phases of the pandemic. We included two triggers that would cause both the general population and university students to increase their adoption of protective behaviors: a high level of COVID-19 patients in critical care and a high number of COVID-19 deaths.

We estimated model parameters, including the duration of time spent in each health state, the infectiousness of COVID-19, the probability of needing general and specialized hospital resources, disease mortality, and the effectiveness of COVID-19 prevention strategies, using the peer-reviewed literature, pre-published reports, and expert opinion (**Table 1**).

We calibrated the model to the observed hospitalization rates and mortality in Middlesex County, Ontario, Canada, which includes the city of London, from March 1 to August 15, 2020. London, Ontario is a medium-sized city that experienced relatively fewer cases of COVID-19 compared to the province of Ontario overall [4] likely due to province-wide response following the detection of community-

transmission in the provincial capital (Toronto) occurring before widespread community transmission began in smaller urban centers. As a result, it is a representative case study of a medium-sized city with relatively few cases in the spring of 2020 facing a major shift in its COVID-19 risk profile in fall with the return of in-person instruction at two large destination post-secondary institutions [5, 6].

In this Supplemental Methods, we provide additional detail on the structural features of the model as well as the identification and selection of input parameter values.

POPULATION

We model a mid-sized city of 500,000 year-round residents, of whom 3,500 (0.7%) live in long-term care. We assume that 20,000 undergraduate university students, between the ages of 18 and 24, also reside in the city during the academic year (between September and April). Faculty, staff, graduate students, and professional program students are considered part of the general population.

DISEASE SEVERITY AND CLINICAL RESOURCE NEED

We stratify the infected population into disease severity groups to capture differences in the likelihood of case identification through symptom-based detection and differences in the likelihood of being hospitalized and needing specialized health care resources. The severity distribution describes the distribution of actual infections, regardless of diagnosis.

Asymptomatic infection: We estimated the proportion of asymptomatic cases to be 31% for both the general population and university students based on an ongoing meta-analysis of reports that include follow-up monitoring for the development of symptoms among asymptomatic cases [7]. University students may have a higher rate of asymptomatic presentation than the general population, but the

literature continues to be very mixed. A follow-up of a cohort of US Navy sailors revealed that only 18% continued to be asymptomatic throughout their full course of infection [8]. However, in a cohort primarily identified as having had COVID-19 via serology, retrospectively 79% of people < 40 years of age could not recall any symptoms [9]. We therefore explored this uncertainty in sensitivity analysis. The proportion of cases that are asymptomatic has also been shown to be correlated with older age; we assumed that 12% of infections in LTC residents are asymptomatic based on follow-up monitoring in a LTC cluster [10, 11].

Symptomatic and not hospitalized infections: For each population, COVID-19 infections that were not classified as asymptomatic, severe, or critical were categorized as mild or moderate. Infections with mild and moderate symptoms are not hospitalized but may present to medical care for assessment and may receive some outpatient care not specifically accounted for in our analysis. In total, 60.4% of the general population and 67.8% of university students were classified as having mild and moderate infections.

LTC residents with severe symptoms, for which they would be considered for hospitalization, may choose to receive limited interventions in favour of supportive care at their LTC facility. Among LTC residents, 76.2% were classified as symptomatic but not hospitalized.

Severe and critical infections: In our model, critical patients are those who require mechanical ventilation (MV) or renal replacement therapy (RRT) in a critical care unit. Severe patients receive hospitalization but do not require MV or RRT.

We estimated the rate of critical care use in the general population and university students based on rates of critical care use among residents of Lombardy Italy, identified by contact tracing, who were either diagnosed with COVID-19 during their infection or identified retrospectively by serology testing [9]. For the general population, we estimated the rate of critical care using the observed rate in 2,666

patients < 80 years of age as 1.7% (95% CI: 1.2, 2.2). For university students, we estimated the rate of critical care using the observed rate in 835 patients < 40 years of age as 0.24% (95% CI: 0, 0.6) [9].

Among critical care patients, we estimate the ratio of patients requiring RRT to MV based on the UK Intensive Care National Audit and Research Centre (ICNARC) report describing the care and outcomes of 10,118 critical care COVID-19 patients in the UK. In this report, 7,277 patients required MV and 2,673 required RRT, resulting in a ratio of 0.37 RRT patients per MV patient [12]. Although the report describes patients in need of both technologies, we did not include this possibility in the model.

Among the 10,137 COVID-19 hospitalizations in Canada between February 23 and June 21, 20.3% involved a critical care admission [13], resulting in an estimated a ratio of 3.92 patients requiring hospitalization without critical care per critical care patient. This is similar to rates of critical care observed in the UK, in which approximately 22% of all hospitalized patients required critical care [14]. Overall, hospitalization rates are lower in younger people, but the fraction of hospitalized cases requiring intensive care appears similar and so we also assumed this ratio of hospitalization without critical care per critical care patient also applied to university students. For example, a US report on 3,222 hospitalization for COVID-19 aged 18-34 years reported 21% requiring intensive care [15]. This led to an initial estimate of 1.0% of infections requiring hospitalization but not critical care in university students (0.24% × 3.92) and 6.7% of infections requiring hospitalization but not critical care in the general population (1.7% × 3.92). The estimated rate of hospitalization in the general population resulted in a higher number than observed in London, Ontario, during model calibration and so the probability of hospitalization without critical care was lowered to 3.75% in the general population in order to better fit the observed local data.

Based on 699 documented COVID-19 cases in LTC residents across Canada between February 23 and June 21, we that assumed 0.3% (95% CI: 0, 0.7) of patients would require critical care and 11.4% (95% CI:

9, 14) would require hospitalization without critical care [13]. LTC residents have a lower rate of critical care use than the general population because LTC residents in medical need of critical care may have directives in place instructing different levels of care.

CLINICAL OUTCOMES AND CLINICAL RESOURCE USE

A schematic of the health states and transition times for infected individuals is presented in **Appendix Figure 1**.

We assumed a mean incubation period, the time from exposure to symptom-onset, of 5.6 days (observed median of 5.1 days [95%CI 2.2, 11.5]) [16]. We divide the incubation period into two sub-states: ‘Exposed, not Infectious’, a pre-infectiousness phase in which the person is exposed and will develop the infection, and ‘Exposed, Infectious’, in which infectious individuals are pre-symptomatic. This two-stage approach is important because an estimated 44% of transmissions occur prior to symptom onset [3]. He et al. estimated infectiousness starts 2.5 days prior to symptom onset, peaks at 0.6 days prior to symptom onset, and declines within 7 days of symptom onset [3]. This is consistent with estimates based on cohorts from Tianjin and Singapore which indicate transmission beginning 2.5 to 2.9 days prior to symptom onset [2]. In our base case analysis, the ‘Exposed, not Infectious’ state has an average duration of 3.1 days and the ‘Exposed, Infectious’ state has an average duration of 2.5 days. In total, we estimate the average duration of infectiousness in asymptomatic and mild or moderate cases to be 10 days [3, 17, 18]. Among individuals who ultimately develop severe or critical symptoms requiring hospitalization, we assumed an average 5.8 days from onset of initial symptoms to the presentation of severe symptoms [14], during which time these patients would be infectious but in home isolation if aware of their infection status.

We assumed that severe patients who do not need critical care (MV or RRT) have an average hospital length of stay of 8.3 days based on length of stay in UK hospitals [14]; critical patients who require invasive mechanical ventilation have a median length of stay of 13 days (IQR: 7, 23) in the critical care unit followed by a median of 7 (IQR: 4,13) days in the hospital [12]; and critical patients who require RRT have a median length of stay of 21 days (IQR: 9, 41) [12]. To ensure realistic distributions for the duration of infectiousness and the duration of hospital resource utilization (e.g., gamma distributions instead of exponential distributions), states were further subdivided into successive otherwise identical states [19, 20].

In the model, all people who survive infection move to a recovered state. We assume that a non-infectious period of home isolation continues for an average 10 days after people are no longer infectious, consistent with discharge instructions [21]. In the model, people with asymptomatic or mild and moderate infections who are not diagnosed during their infectious period transition into the recovered health states in which they continue to participate in physical distancing consistent with rates in the susceptible population as they will continue to adhere to behaviours consistent with individuals who believe themselves not to have been infected.

MORTALITY

We estimated mortality rates for critical care patients, stratified by age category, using the UK ICNARC report describing the care and outcomes of 10,118 critical care COVID-19 patients in the UK [12]. We estimated mortality rates for patients treated in hospital, but not in critical care, using a report of over 20,000 hospitalized patients in the UK [14], using the < 55 years of age population to estimate mortality in university students (0.43%) and the < 70 year old population to estimate mortality in the general population (14.4%). For LTC residents who are and are not hospitalized, we estimated the infection

fatality rate based on the observed outcomes in 680 Canadian LTC patients to be 47.4% and 25.5%, respectively [13], For the general population and university students, we assumed that there was no mortality risk for individuals with asymptomatic infections and infections not requiring hospitalization.

If, due to capacity constraints, patients receive a lower level of care than is medically indicated, we assumed higher mortality rates based on expert opinion. For patients with severe symptoms requiring hospitalization who are instead isolating at home, we assumed a 25% case fatality rate. For patients in hospital who need but are unable to access critical care (either MV or RRT), we assumed a 40% daily probability of death, resulting in a life expectancy of approximately 2 days. For patients in home isolation who need critical care (either mechanical ventilation or RRT) but are unable to access either critical care or hospitalization, we assumed a 60% daily probability of death, corresponding to a life expectancy of approximately 1 day.

We did not include mortality from causes other than COVID-19 in the model.

HEALTH CARE RESOURCE CAPACITY

Consistent with the average of 14.2 per 100,000 population in Ontario [22], normal pre-COVID critical care capacity of our center is 69 beds [23]. In a crisis situation, reductions in other services, secondment of staff from other units, and accessing strategic stockpiles of equipment [24], can enable short-term expansions in capacity. In our centre, reductions in other services can facilitate operation of up to 184 critical care beds [23]. In the base case, we estimated hospital acute care, MV, and RRT capacity of 500 beds, 120 beds, and 30 RRT patients was the maximum amount that could be allocated to COVID-19 patients. If these thresholds are exceeded, COVID-19 patients are directed to the next-best available care often facing higher mortality rates. In the model implementation, each day, we first calculated the new demand for hospital resources and then allocated patients to beds depending on whether the total

demand exceeded available capacity. For example, if there was more demand for ICU beds than was available, patients were allocated to a hospital ward bed if one was available. If there were no available hospital ward beds, patients were not admitted to the hospital.

In discussion of results, we focus on a threshold of 30 critical care beds for COVID-19 patients as a local policy-relevant threshold necessitating the partial closure of non-COVID-19 health services. Based on expert opinion, substantial reductions in the provision of other types of health care (such as cancelling elective surgeries) will be required if 30 critical care beds are occupied with COVID-19 patients.

DIAGNOSIS BY CLINICAL PRESENTATION AND CONTACT TRACING

The time from symptom presentation to diagnosis in symptomatic cases decreased over the course of the pandemic with increased awareness of the disease and its varied range of symptoms [25]; in the base case analysis we assumed the minimum time from symptom onset to diagnosis to be 2.1 days consistent with the minimum time to self-assess, seek medical attention, and receive diagnostic results [26]. The observed median time to diagnosis through symptom-based surveillance alone of 4.6 days (95%CI: 4.2, 5.0) and symptom-based surveillance in combination with contact tracing efforts of 2.9 days (95%CI 2.4, 3.4) [27]. From this, we estimated that symptom-based surveillance and contact tracing results in a daily probability of diagnosis of 15.8% in individuals with symptoms and the daily probability of detection from contact tracing of 4.1% in asymptomatic infections. This combination of assumptions resulted in approximately 22% of infected individuals being identified, consistent with the overall rates of diagnosis implied by preliminary serology data in Ontario [28].

For LTC residents, we assume that twice daily symptom screening results in a 40% daily probability of detection in symptomatic patients and that contact tracing with access to all resident contacts increases that probability to 52%. Contact tracing results in a 8.2% daily probability of diagnosis in asymptomatic

cases which we assume is increased further by routine testing for COVID-19 every 14 days which is recommended for the staff of LTC facilities in Ontario [29]. Universal testing of all residents in a nursing home with a single new case is recommended by the US Centers for Disease Control and Prevention [30]. We assumed this routine testing would be performed using nasopharyngeal swab and PCR analysis with a test sensitivity of 72.1% [31, 32].

CONTACT MIXING PATTERNS

General population: Based on an extrapolation of the 2008 POLYMOD study in Europe to reflect network structure of the Canadian population, the average number of contacts per person in Canada is 12.6 per day [33]. Of these contacts, on average, 1.0 contacts are with those aged 20-24 [33]. Nationally, 6.6% of the Canadian population is aged 20-24. We assumed that the age distribution of the year-round residents in our simulated city matches that of Canada nationally. However, during the academic year, we assumed that the 20,000 university students consist entirely of 20-24 year-olds. Thus, during the academic year, university students in our simulated city comprise 38% of all 20-24 year-olds in the overall community (20,000 / (500,000 × 6.6% + 20,000)). As a result, we estimated that of the 1.0 daily contacts that the general population has with the 20-24 year-old age group, 0.38 contacts per day would be with university students when the university is in session.

The average number of daily contacts that a person in the general population has with someone in LTC was calculated in order to balance the total number of daily contacts between LTC residents and visitors/staff (who are considered to be from the general population). With 3,500 LTC residents each with 13.7 staff contacts and 0.48 visitor contacts per day, this implies a total of 49,630 contacts per day between LTC residents and the general population (3,500 × (13.7+0.48)). Dividing by the size of the

general population (500,000 – 3,500 = 496,500), this yields a total of 0.1 LTC resident contacts per person in the general population per day.

University students: Compared to the average number of contacts for the overall population, Canadians aged 20-24 have higher number of close contacts per day (15.7 vs. 12.6) [33]. Further, using self-report diaries, several studies specifically of university students report still higher numbers of contacts per day than the average person in their age demographic. For example, a convenience sample from two British universities reported an average of 21.9 contacts per day on weekdays and 14.5 contacts per day on weekends for participants aged 20 to 29 [34]. A study at the University of Antwerp, in which 83% of study participants were students, reported an average of 23.7 contacts per day [35]. A study of 28 students at the University of Warwick reported 26 contacts per day during the week and 19 contacts per day during the weekend [36]. A German study of 556 first year university students reported an average of 8.9 direct conversational contacts per weekday and 12 direct conversational contacts on weekend days; expanding to also report contacts in large groups (e.g., in a lecture hall) or random encounters within 2 meters (e.g., on public transit), the average number of contacts per day increased to 47 and 62 contacts per day, respectively [37]. Compared to studies using self-report diary design, studies with electronic detection of interactions identify higher contact rates in school environments. A study of interactions at a US high school in which 94% of students, staff, and teachers were tagged with wireless sensors to record contacts indicated an average of 50 contacts within 3 meters of greater than 10 min in duration and an average of 30 contacts within 3 meters with greater than 20 minutes in duration within a school day [38]. Similarly, a study in which university students living across six residence falls were provided devices to monitor Bluetooth interactions with other devices in the study and other devices with "discoverable" Bluetooth (within 5 meters) indicated a total of 219 contacts per phone per day [39].

In our base case analysis, we assume that students have 23.7 contacts per day, based on a study at the University of Antwerp [35]. We assumed 60% of those contacts are with other university students based on the age distribution of student's reported contacts [34, 35], with the remainder being with members of the 'general population' which includes staff and faculty of the university as well as other members of the community when students are in transit, shopping, and working in jobs in the community or non-student members of their household. This number of contacts between university students and the general population (9.48 contacts per day) balanced with the estimated number of contacts the general population has with university students each day (20,000 × 9.48 / 496,500 =0.38). Several of the studies estimating contacts among university students were performed in winter, and so may underestimate the number of contacts students have during the early weeks of the new academic year. We explore this uncertainty in scenarios with higher contact rates for a short period of time upon arrival to campus on September 1.

Long-term care residents: Based on a Canadian study in which a sample of long-term care residents and staff were equipped with RFID tags in order to estimate the number of contacts, we estimated the number of resident-resident and resident-staff contacts [40, 41]. Adjusting for the likelihood of observing a contact (which required both individuals to be participating in the study), we estimate that there are 19.9 resident-resident contacts per day (95% CI: 11.3 – 28.5) and 13.7 resident-staff contacts per day (95%CI: 11.4 – 15.9) using the summary participant data provided by the study's authors (personal communication: S. Moghadas). This study did not capture resident-visitor contacts; we assumed 0.48 visitors per day based on the distribution of visit frequency in the 2012 Ohio Nursing Home Family Satisfaction Survey [42]. Thus, in total, LTC residents have 14.2 contacts with the general population each day. We assumed no contact directly with university students.

DISEASE TRANSMISSION

Using exponential regression, we empirically estimated the basic reproduction number, R_0, the average number of secondary cases produced by one infected individual during the infected individual's entire infectious period assuming a fully susceptible population, is 3.0 based on Ontario's reported cases between March 7 to March 22 [43]. Using an average duration of infectiousness of 10 days and an average number of close contacts per person of 12.6 [33], we calculate the probability of transmission between a susceptible and an infected person, in the absence of any interventions, to be 0.024. We also considered using an R_0 of 3.5, estimated for Ontario using a date range of March 8 to 14 [44]. The initial cumulative infection curve in Ontario was dominated by cases in Toronto which has high population density in comparison to London and other mid-size communities with university student populations that are relatively large compared to the population of the community. Also, an R_0 of 3.0 demonstrated greater fit to the initial trajectory of the infection in the calibration step.

In the model, we assume a 90% reduction in contacts for people who are aware of their infection status and in home isolation, which is at the high end of observed adherence to quarantine instructions in past epidemics [45, 46]. We assume isolation of hospitalized patients is 100% effective at preventing transmission to others.

Each day, we calculated the number of new infections from contacts between susceptible people in Group i, S_i, where

$$i = \{General\ population, lowPD;\ General\ population, highPD;\ University\ students, lowPD;$$

$$University\ students, highPD;\ LTC\ residents, lowPD;\ LTC\ residents, highPD\}$$

with people in Group j, where $j = \{General\ population, University\ students, LTC\ residents\}$ to be

$$\dot{I_{i,j}} = S_i \left(1 - \beta \sum_k \frac{I_{j,k}\left[1 - \kappa_{j,k}\right]\varepsilon_{ijk}}{\sum_k N_{j,k}\left[1 - \kappa_{j,k}\right]} \right)^{C_{ij}[1-\kappa_i]}$$

where k represents subpopulations within Group j with different prevalence, contact behaviours, and mask wearing behaviours (i.e., $k = Unaware\ lowPD, Unaware\ highPD, Aware\ Asymptomatic,$ $Aware\ Symptomatic, Aware\ Isolation\ Home, AwareIsolationLTC\}$); $I_{j,k}$ represents the number of infected people in Group j subgroup k; $\kappa_{j,k}$ represents the reduction in contacts for people in Group j subgroup k; ε_{ijk} represents the relative reduction in transmission due to mask wearing behaviours; $N_{j,k}$ represents the number of individuals in Group j subgroup k; C_{ij} represents the number of contacts between people in Group i and people in Group j; κ_i represents the reduction in contacts for people in Group i; and β represents the probability of transmission conditional on contact between a susceptible and an infected individual. The relative reduction in transmission due to mask wearing is calculated as

$$\varepsilon_{ijk} = (1 - M_i)\left(1 - M_{j,k}\right) + M_i\left(1 - M_{j,k}\right)(1 - \theta_S) + (1 - M_i)M_{j,k}(1 - \theta_I) + M_i M_{j,k}(1 - \theta_S)(1 - \theta_I)$$

where M_i represents the proportion of Group i wearing a mask with their remaining contacts; $M_{j,k}$ represents the proportion of Group j subgroup k wearing a mask with their remaining contacts; θ_S represents the effectiveness of a mask when worn by the susceptible individual; and, θ_I represents the effectiveness of a mask when worn by the infected individual. For hospitalized health states, we assume a 100% contact reduction, so hospitalized patients do not contribute to transmission.

INITIAL COMMUNITY BEHAVIOUR

General population: An Angus Reid poll of Canadians, taken in the first week of August, classified COVID-19 prevention behaviours of Canadians into three groups: “Infection Fighters”, “Inconsistent”,

and “Cynical Spreaders” [47]. In part based on this stratification, we divided the general population into two risk groups based on their intensity of prevention behaviours. One of those risk groups (‘high-intensity physical distancers’) engage in high levels of risk mitigation behaviours (similar to “Infection Fighters”), while the other has low uptake of prevention behaviours (similar to the “Cynical Spreaders”). In calculating the prevention behaviours (level of contact reduction and mask wearing) from the Angus Reid poll for the risk groups in our model, we split the “Inconsistent” group evenly between the “Infection Fighters” and the “Cynical Spreaders”.

In the base case, we assumed that 40% of the general population are initially ‘high intensity physical distancers’ consistent with 47% of Canadians reporting “Infection Fighter” behaviour in the Angus Reid poll [47]. We assume that ‘high-intensity physical distancers’ reduce their average number of contacts by 75% (from 12.6 to 3.2 contacts per day) consistent with 70% of “Infection Fighters” reporting fewer than 5 contacts outside of their household, and that they wear a cloth mask with 86% of their remaining contacts [47]. Due to reduced density in public spaces and reduced availability of their usual contacts, the remainder of the population also experiences an overall contact reduction calculated at each time to be equal to the reduction in overall contacts imposed by the ‘high intensity physical distancers’ [Proportion of the population that are ‘high intensity physical distancers’ × 75% reduction in contacts]. Further, we assume that these ‘low intensity physical distancers’ are using a cloth mask with 38% of their contacts [47]. We assume the effectiveness of cloth masks in reducing disease transmission is 40% based on a German study using synthetic control to evaluate the effectiveness of real-world mask use [48].

University students: During the COVID-19 pandemic, many university-aged persons report taking COVID-19 infection precautions, but to a lesser extent than other age groups [47]. The responses of 18-34 year-olds to the recent Angus Reid COVID-19 behaviour survey indicated that 60% have more than 2

COVID risk behaviours (compared to 30% in the population overall); in addition, 16% report having 10-19 contacts and 14% more than 20 contacts outside their household [47].

In the base case, we assumed that, on average, university students reduce their contacts by 40% (from 23.7 to 14.1 contacts). This was to approximate the net behaviour of 18-24 year-olds in the Angus Reid survey, of which 32% reported “Infection Fighter” behaviour, which we equate with a 75% reduction in contacts in the general population and the remainder experiencing a reduction in contacts due to reduced access to their usual contacts. Thus, overall the net contact reduction was calculated as 32% × 75% + (1 – 32%) × (32% × 75%) = 40.3%. In this same survey, 57% of 18-24 year-olds report wearing a mask indoors with people outside their household [47], so we assumed this level of mask wearing among all university students.

Long term care: The ability to reduce contacts in LTC facilities is limited by multiple occupancy rooms and the requirements of staff to provide medical care and support with activities of daily living. However, reductions in contacts can also be used as a proxy for contacts protected by highly effective medical personal protective equipment donned and doffed by trained personnel. We assume that LTC residents have a 50% reduction in their average contacts (from 34.1 contacts to 17.05 contacts). We further assume that of their remaining contacts, 86% are protected by cloth masks providing a 40% reduction in disease transmission [48].

COMMUNITY BEHAVIOUR CHANGES IN RESPONSE TO COVID-19 OUTCOMES

We assumed that the population responds to COVID-19 outcomes in the community, specifically reduced access to non-COVID health services and COVID-19 mortality, by increasing their protective behaviours, as observed in a US-based study [49]. These changes in behaviour are intended to capture both individual decision-making and policy changes that may be instituted by the city.

We relied on locally relevant thresholds to inform triggers for community behaviour change. Based on expert opinion, substantial reductions in access to other health care services would need to occur if 30 critical care beds (about 40% of normal critical care capacity in a city of 500,000 [22]) were occupied by COVID-19 patients. Therefore, we set one of the responsive behaviour triggers to activate when there are 15 COVID-19 patients in critical care, representing 50% of the capacity available to COVID-19 patients without modifying access to other health care services.

General population: We assumed that proportion of people who are 'high intensity physical distancers' increases by 0.5% each day if the number of COVID-19 patients in critical care exceeds 15 and by an additional 1.0% each day if the number of COVID-19 deaths in the past 10 days exceeds 10. In the base case, we assume that a maximum of 80% of the general population can become 'high intensity physical distancers,' reflecting the fact that not all individuals are able or willing to engage in physical distancing and high levels of mask wearing. To incorporate the re-opening and relaxation of behaviour that occurs when the prevalence of COVID-19 is low, we also assumed that proportion of people who are 'high intensity physical distancers' decreases by 0.5% each day if the number of COVID-19 patients in critical care is fewer than 10 and by an additional 1.0% each day if the number of COVID-19 deaths in the past 10 days is fewer than 5.

University students: We assumed that university students respond to the same triggers as the general population and increase and decrease their physical distancing behavior at the same rate, up to a maximum of a 75% reduction in contacts per day (23.7 contacts reduced to 5.9 contacts per day).

Long term care: Social interaction for residents such as communal meals and recreational activities, and visits with family are important for resident wellbeing. However, we assumed that more intensive restrictions on these activities would be re-instituted if adverse COVID-19 outcomes begin to appear in the community. While LTC facilities may receive their direction from provincial decision makers, we

assumed that their COVID-19 precautions would also change with local outcomes consistent with the responsive behaviour of the general population. We assumed that the reduction in contacts increases by 0.5% each day if the number of COVID-19 patients in critical care exceeds 15 and by an additional 1.0% each day if the number of COVID-19 deaths in the past 10 days exceeds 10. We assume the maximum reduction in contacts is 80% (from 34.1 contacts to 6.8 contacts per day) and that 86% of remaining contacts are protected by a cloth mask.

MODEL CALIBRATION

We estimated some model parameters using calibration to the observed critical care and ward hospitalizations at London Health Sciences Centre, LTC resident mortality, and overall mortality in London-Middlesex Ontario from March 1 to August 15, 2020 (**Appendix Figure 2**). All non-essential workplaces were ordered closed on March 23 at which time, in the model, individuals began reducing their contacts [50]. We used trends in cell phone mobility data [51, 52] to inform estimates of the proportion of the population participating in high-intensity physical distancing over time (**Appendix Figure 3, Appendix Figure 4**). Further, beginning May 16 [53], we assumed that people engaged in high-intensity physical distancing wore a mask for 86% of their remaining contacts and that people not engaged in high-intensity physical distancing wore a mask for 38% of their contacts, consistent with the levels used in our policy analysis.

To fit to the calibration targets we tuned the R_0 parameter, selecting 3.0 rather than 3.5 because of the better fit to the London Ontario data, which we also believe would be representative of mid-sized, relatively lower-density cities.

We also tuned the proportion of the general population who would be in need of hospitalization. Based on the ratio of critical care to non-critical care hospitalizations estimated from the literature, described

above, we had initially estimated the value to be 6.8%, but this number resulted in an overestimate of ward hospitalizations when compared to the London, Ontario observational data. We found that 3.75% represented a closer fit to the data.

Finally, we did not find an estimate for the length of time until recovery or death for a COVID-19 patient receiving care in a LTC facility. We estimated this parameter to be 18 days (median 12.5 days) fitting the shape of the mortality curve for LTC residents.

After June 7, 2020, there were fewer than 5 patients in critical care and nearly always fewer than 5 patients in ward hospital beds in London hospitals which is the minimum number for reporting. There were also few new infections reported by the Health Unit. Over the month of July, the number of active cases decreased from 62 on July 1 to 32 on July 31; there were 28 active cases on August 15 [54]. At the end of the calibration window, we selected different levels of physical distancing behaviour to create different starting conditions for analysis. For the base case, we selected the proportion of the population engaged in high-intensity physical distancing beginning in July to be 40% consistent with the which resulted in 40 active infections (some of which would be undiagnosed) on August 15. By selecting different values for the proportion of the population engaged in high-intensity physical distancing in July, we created alternative scenarios in which there were 25, 50, and 75 people with active infections on August 15 to evaluate as alternative scenarios in sensitivity analysis.

MODEL INITIALIZATATION

In the base case, at the start of the simulation, there were 40 infected individuals, some undiagnosed, in the general population. We assumed that there are no COVID-19 infections among LTC residents. The actual number of diagnosed active cases on August 15 in London-Middlesex was 28 (based on data retrieved daily from the London Middlesex Health Unit webpage [54]).

The 40 cases of COVID-19 were distributed across the COVID-19 health states based on state distribution of the population at the end of the run-in period of the model, March 1 to August 15. On August 15, the population is distributed as follows: 493,751 Susceptible, 12.5 Exposed but not yet infectious, 38.3 infected individuals (10.0 exposed and infectious, 7.9 asymptomatic individuals, 17.3 mildly symptomatic individuals, 1.2 symptomatic individuals who will progress, but have not yet progressed, to needing hospitalization, and 1.9 patients in hospital), 2,663 Recovered individuals, and 35 individuals in the general population who died from COVID-19 between March 1 and August 15. The cumulative number of diagnosed cases in London-Middlesex on August 15th was 707 [54], which implies an overall detection rate that is consistent but somewhat higher than the estimate of 22% case detection rate the end of June in Ontario based on blood sample serology [28].

Based on the state distribution of LTC residents on August 15 as estimated through the model run-in period, there were 83 residents in the recovered health states at the start of the simulation. Over the course of the run-in period, 27.7 deaths occurred in LTC facilities (consistent with the reported number of 24 deaths in LTC residents by July 31 in London-Middlesex [54]); we assume that new residents moved in resulting in a state distribution of LTC residents on August 1 of 3417 Susceptible and 83 Recovered LTC residents.

ANALYSIS OF UNCERTAINTY AND POLICY ALTERNATIVES

We used the model to explore how COVD-19 risk and prevention behaviours of the general population and the student community, in particular, affect the incremental COVID-19 burden attributable to the arrival of 20,000 university (post-secondary) students on September 1st.

Under different scenarios of community physical distancing effort, student behaviour, and routine testing in students, we calculated the number of infected individuals, demand hospital resources, time to responsive behaviour thresholds being activated, and health outcomes overall and over time.

Evaluating the incremental impact of university student behaviour on community covid-19 outcomes

We considered several city scenarios varying the following city features

- Different levels of general population participation in high-intensity physical distancing,
- Different initial numbers of infected cases in the population,
- Different thresholds for engaging greater participation in physical distancing,
- Different intensity of response after crossing the trigger thresholds.

Evaluating the uncertainty in short-term student behaviour

We considered several levels of student COVID-19 prevention behaviours

- Different levels of immediate contact reduction,
- Delay in the initiation of contact reduction for a short-term period at the beginning of the school year,
- Short-term increase in the number of contacts before initiating a reduction contacts.

We also performed extensive sensitivity analysis on other parameters

- The number of students with COVID-19 infection when they arrive on campus,
- Intensity of student response to behaviour thresholds,
- Increases and decreases to student mask wearing.

Comparing frequencies of routine diagnostic testing targeted at the student population

Finally, we calculated the number of infections averted through routine and one-time screening of university students. We considered routine screening of students on short periodic intervals (every 28 days, 14 days, 10 days, 7 days, 5 days, and 3 days). We also considered a one-time screening of all university students on September 22 (three weeks after the arrival of students). We assumed routine testing would be performed using nasopharyngeal swab and PCR analysis with a test sensitivity of 72.1% [31, 32].

Economic analysis

High-frequency testing requires substantial resources with financial implications. In order to compare the cost of testing against its value, we estimated the costs of averted COVID-19 hospitalizations and we converted averted COVID-19 deaths into monetary benefits using a health economic framework. We also estimated the productivity costs associated with infections. As a simplification, we did not include other benefits of averting COVID-19 infections, such as potential long-term complications and their associated reductions in quality of life, and the community economic benefits of delaying social and economic restrictions. Future work is needed to quantify the full cost and quality of life impacts of COVID-19.

When evaluating the economic value of life years lost due to premature death, we considered a willingness-to-pay of $50,000 and $100,000 per life-year gained, which is a commonly used threshold in in Canada [55, 56] and the US, respectively [57, 58]. We calculate the number of life-years gained by estimating the average remaining life-expectancy of patients succumbing to COVID-19. We estimate the age distribution of those dying from COVID-19 based on the distribution reported by the US Centers for Disease Control and Prevention [59]. We estimate mortality in the absence of a COVID-19 infection using

the Canadian lifetables for 2016 to 2018 [60] and discount life-expectancy by 1.5% per year, consistent with Canadian health economic evaluation guidelines [55]. We calculate the average discounted life-years lost to be 12.2 years and so a total value of $610,075 per death averted at a willingness-to-pay of $50,000 per life-year gained. At the higher willingness-to-pay threshold of $100,000 per life-year gained, the average value is $1,220,150 per death averted. These values are lower than other estimates of the economic value of a COVID-19 death averted [61] because we have selected a health economic framework consistent with how health technologies are evaluated and compared in Canada.

The average daily cost of ICU and general ward hospitalization in Canada is estimated at $3,592 and $1,135 (in 2013 Canadian dollars) [22]. Using the Canadian GDP deflator to adjust for inflation [62, 63], the estimated daily cost of ICU and general ward hospitalization is $3,970 and $1254 in 2020 Canadian dollars [22]. Multiplying by the average length of stay for a COVID-19 patient admitted to the ICU (15.5 ICU days and 10.1 ward days) results in an average cost of $74,157 per ICU admission. Similarly, multiplying by the average length of stay for a COVID-19 patient admitted to the hospital, but never requiring ICU care (8.3 days), results in an average cost of $10,411 per admission.

To estimate the average cost of lost productivity from incremental infections, we used the average weekly income in Canada of $1,045 in 2020 Canadian dollars (reported by Statistics Canada to be $1028.50 in 2019 dollars [64]). We estimate the productivity loss by assuming that infected individuals lose two-weeks of productivity. This approach may over or underestimate the direct productivity loss of an infected individual. Not all infected individuals will be diagnosed with COVID-19 and required to isolate, but at least some undiagnosed symptomatic individuals may still stay home from work. Conversely, among diagnosed individuals, multiple individuals in their household may be required to isolate even though they may not become infected. At a population level, this approach may also underestimate total productivity costs per infection because it fails to capture the indirect costs of each infection’s contribution to increasing social and economic restrictions.

SUPPLEMENTAL RESULTS

City epidemic outcomes without the introduction of the university student population

Without the introduction of the student population, the base case assumptions for the general population and LTC residents leads to a total of 3,515 infections over 4.5 months (August 15 to December 31). In this scenario, infections, hospitalizations, and daily deaths do not peak until the new year (**Appendix Table 1**). Demand for critical care (mechanical ventilation or renal replacement therapy) peaks at 25 beds early in the new year, and a total of 37 COVID-19 deaths occur between August 15 and December 31 (78 deaths by January 31, 2021). The timing and magnitude of the city's COVID-19 outbreak, excluding any impacts from students, is determined by the initial number of COVID-19 infections in the community, the level of participation in physical distancing, the responsiveness of the community to increasing critical care cases and COVID-19 deaths, and the proportion of contacts that are protected with mask wearing (**Appendix Figures 5-7**).

Impact of students arriving with infections

If some students arrive exposed or asymptomatically infected, the total number of infections occurring over the course of the semester increases. For example, if 10 students arrive infected, the number of infections increases by 5,419 over the base case. The impact of students arriving already exposed or infected in the community is most substantial on the timing of peak infections, peak hospitalizations, peak critical care utilization, and the timing of responsive behaviour triggers. Compared to the scenario without the introduction of the student population, responsive behaviours are triggered 5.6 weeks earlier if 10 students are infected when they arrive (**Appendix Table 3**).

Sensitivity analysis

We performed extensive sensitivity analysis exploring the impact of general population and student population COVID-19 prevention behaviours on the incremental impact of introducing students into the community.

The negative impacts of introducing the student population can be partially mitigated through high uptake of COVID-19 preventive behaviours in the student population including high rates of contact reduction or if the rate of mask wearing significantly exceeds the level reported by 18 to 24 year-olds (**Appendix Figure 10, Appendix Table 6**). For example, if students immediately reduce their contacts by, on average, 50% (from 23.7 to 11.9 contacts per day) and wear masks to protect 65% of their remaining contacts, the incremental number of infections attributable to the arrival of the student population can be reduced to 1,807 infections (from 3,515 to 5,322), representing only a 51% increase over the number of infections the community would expect without the students, and delays the activation of responsive behaviour triggers by 1 week.

The magnitude of the impact of the introducing the student population is also determined by the COVID-19 prevention behaviours of the general population. Counter-intuitively, the relative impact of introducing the student population is greatest when the prevention efforts by the general population are high (**Appendix Figure 11**). For example, when 50% of the general population are participating in high-intensity physical distancing, without students the number of new infections per day is nearly constant over time, resulting in a very low level of cumulative infections over the semester (total of 649 infections). Introduction of the students results in 1,274 additional infections, doubling the total number of infections expected in the city without the addition of the student population (**Appendix Table 2**). In such a scenario, because the student population is an important determinant of city outcomes, the impact of routine COVID-19 screening in the student population is greater (**Figure 3C**).

Conversely, when only 30% of the of the general population are participating in high-intensity physical distancing, the number of new infections per day is increasing over time even without the students, resulting in 9,277 cumulative infections over the semester. Introduction of the students results in 3,786 additional infections, a 41% increase in the total number of infections expected in the city without the addition of the student population (**Appendix Table 2**). In this scenario, relatively low rates of self-protective behaviour in the general population are the primary driver of community COVID-19 outcomes. Consistent across all scenarios varying the physical distancing behaviour of the general population, is the finding that the number of general population infections that are attributable to the return of the student population is about twice the number that occurs in the student population itself.

Economic analysis

Our evaluation of testing frequency identified the expected number of COVID-19 deaths averted under various testing frequencies and different assumptions of student initial behaviour (**Table 2**). For example, testing every 5 days under the base case assumption that students engage in physical distancing behaviour immediately leads to an expected 1,685 infections, 19.3 critical care admissions, and 15.0 COVID-19 deaths averted. This translates to an economic value of $14.6 million to $23.7 million using a willingness-to-pay of $50,000 and $100,000 per life-year gained, respectively (**Appendix Table 7**). Testing every 5 days would require 4,000 tests per day for approximately 100 days. Thus, each test provides an economic value of at least $36 to $59; a testing strategy that is able to deliver tests for less than this amount would provide more value than its cost. However, even if test delivery is more expensive than $59 per test, it may still provide a net benefit, as our calculation only accounts for direct costs of COVID-19 health care, mortality, and productivity losses directly associated with infections; in

reality, there are many other benefits of reducing COVID-19 community burden, including the community economic benefits of delaying social and economic restrictions.

If students engage in 2 weeks of double the number of student contacts, a 5-day testing schedule averted an expected 2,873 infections, 31.3 critical care admissions, and 24.3 COVID-19 deaths (**Table 2**), leading to an economic value of $23.9 million to $38.7 million or $60 to $97 per test (**Appendix Table 7**). If students are more likely to be asymptomatic than the general population, the number of deaths averted from a screening program increase further, as does the estimated value per test.

Routine testing to identify and isolate asymptomatic infections for the purposes of reducing community transmission risk requires a large number of tests each day and may strain community testing resources. We also evaluated the benefits of a one-time universal screening event occurring three weeks after the students arrive. Compared to routine testing every 5 days, which would require more than 400,000 tests to be performed over the semester, this strategy would only require 20,000 tests. In the case that students double their contacts with other students for a period of two-weeks, this strategy prevents 290 infections, 3.2 critical care admissions, and 2.5 COVID-19 deaths (**Table 2**). This leads to an expected economic value of $2.5 million to $4.0 million using a willingness-to-pay of $50,000 and $100,000 per life-year gained, respectively. Testing once would require 20,000 tests in total, resulting in an economic value of $123 to $199 per test (**Appendix Table 7**). Testing once has a consistently high value per test across scenarios in which students engage in a short-term increase in student contacts but has a relatively low value per test if student behaviour is consistent over the term, even if it is a consistently relatively low level of contact reduction.

Appendix Figure 2 presents a comparison of the modelled outcomes to the observed data, including hospitalization data and COVID-19 mortality, from the City of London and Middlesex County, Ontario, Canada from March 1 to August 15.

Appendix Figure 3 presents assumptions around engagement in physical distancing and mask wearing behaviour between March 1 and August 15 used in the calibration of the model.

Appendix Figure 4 presents the Google mobility data for Middlesex County (including the City of London) from March 1 to August 31 which was used as one source informing the physical distancing assumptions in the calibration of the model.

Appendix Figures 5 though 7 present the number of new COVID-19 infections per day, critical care demand, and COVID-19 mortality between August 15 and December 31 without the arrival of the student population varying the number of COVID-19 infections in the city on August 15 and the level of participation in high-intensity physical distancing going forward.

Appendix Figure 8 presents the cumulative COVID-19 mortality between August 15 and December 31 varying the initial level of student engagement in reducing physical contacts.

Appendix Figure 9 presents the number of new COVID-19 infections per day in the general population (panel A) and in the student population (panel B) between August 15 and December 31 varying the initial level of student engagement in reducing physical contacts and the testing strategy. This figure illustrates that the increase in cases occurs first in the relatively high contact university student population followed by an increase in daily infections in the general population. This figure also

illustrates the effect of routine screening of university students every 5 days and a one-time universal screening event targeted at the university student population on the number of infections per day in the university student population and the general population.

Appendix Figure 10 presents the cumulative number of COVID-19 infections and deaths between August 15 and December 31 with and without the student population. The scenarios along the X-axis present sensitivity analysis on the behaviour of the student population in terms of level of contacts reduction and mask wearing behaviours.

Appendix Figure 11 presents the cumulative number of COVID-19 infections and deaths between August 15 and December 31 with and without the student population. The scenarios along the X-axis present sensitivity analysis on the behaviour of the general population, varying the number of cases in the city on August 15 and the level of general population participation in high-intensity physical distancing.

Appendix Table 1 presents detailed epidemic outcomes *without* the introduction of 20,000 university students. Scenarios consider different initial community conditions and physical distancing (PD) behaviours in the general population (corresponding to results presented in **Appendix Figures 5, 6, and 7**).

Appendix Table 2 presents detailed epidemic outcomes *with* the introduction of 20,000 university students on September 1. Scenarios consider different initial community conditions and physical distancing (PD) behaviours in the general population.

Appendix Table 3 presents detailed epidemic outcomes for sensitivity analysis on the number of COVID-19 infections in the initial student population on September 1.

Appendix Table 4 presents detailed epidemic outcomes for scenarios that vary on initial student participation in physical distancing immediately upon arriving to the community (corresponding to results presented in **Figure 2**).

Appendix Table 5 presents detailed epidemic outcomes for various frequencies of routine testing in university students under different scenarios of initial contact reduction behaviour in students and participation in high-intensity physical distancing behaviour in the general population (corresponding to results presented in **Figure 3**).

Appendix Table 6 presents detailed epidemic outcomes for sensitivity analysis on student population characteristics including initial contact reductions and mask wearing behaviours.

Appendix Table 7 presents detailed breakdown of the economic value associated with infections, hospitalizations, and deaths averted through screening strategies targeted at the university student population.

APPENDIX FIGURE 1. Schematic of the model states for Asymptomatic, Mild and Moderate, and Severe and Critical patients accounting for incubation period, pre-symptomatic infectiousness, delay in diagnosis from symptom onset, and the utilization of hospital resources. To ensure realistic distributions for the duration of infectiousness and the duration of hospital resource utilization (e.g., gamma distributions instead of exponential distributions), states indicated with an asterisk (*) were further subdivided into two successive states splitting equally the average duration of the state.

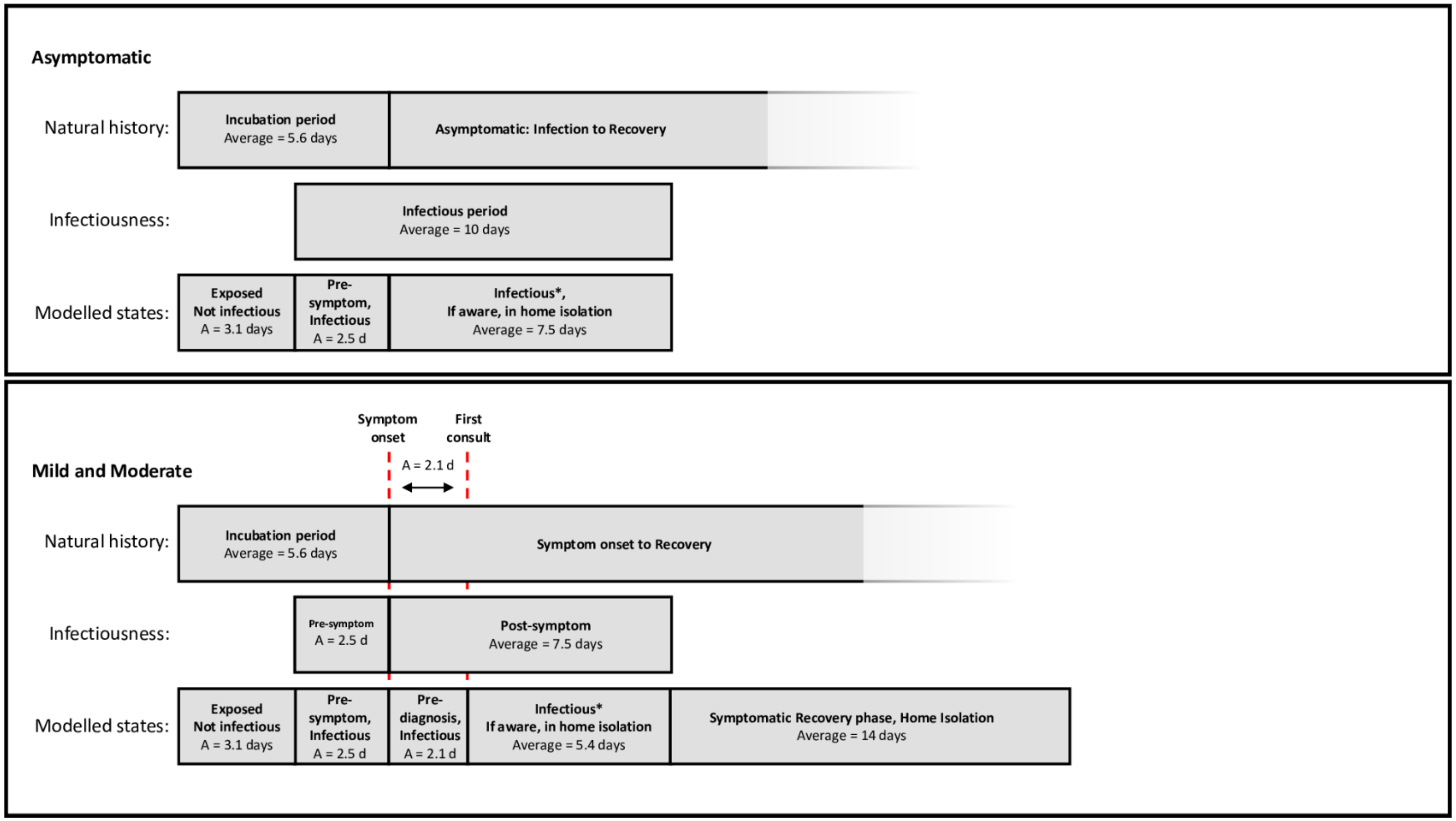

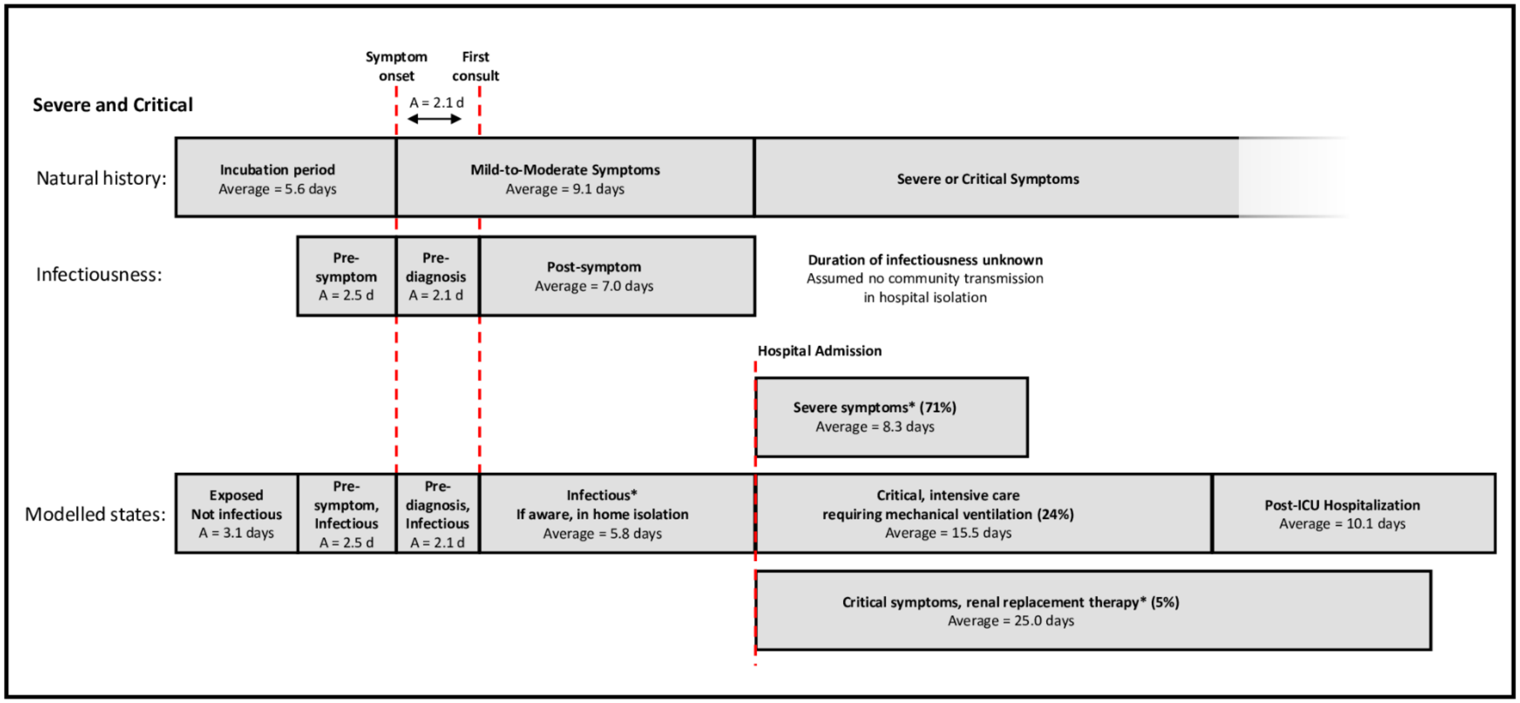
Severe and Critical
Symptom onset
First consult
A = 2.1 d
Natural history:
Incubation period
Average = 5.6 days
Mild-to-Moderate Symptoms
Average = 9.1 days
Severe or Critical Symptoms
Infectiousness:
Pre-symptom
A = 2.5 d
Pre-diagnosis
A = 2.1 d
Post-symptom
Average = 7.0 days
Duration of infectiousness unknown
Assumed no community transmission in hospital isolation
Hospital Admission
Severe symptoms* (71%)
Average = 8.3 days
Modelled states:
Exposed
Not infectious
A = 3.1 days
Pre-symptom, Infectious
A = 2.5 d
Pre-diagnosis, Infectious
A = 2.1 d
Infectious*
If aware, in home isolation
Average = 5.8 days
Critical, intensive care requiring mechanical ventilation (24%)
Average = 15.5 days
Post-ICU Hospitalization
Average = 10.1 days
Critical symptoms, renal replacement therapy* (5%)
Average = 25.0 days

APPENDIX FIGURE 2. Calibration to observed data from London-Middlesex, Ontario, Canada from March 1, 2020 to August 15, 2020. (A) Modelled hospital and critical care occupancy compared to reported COVID-19 hospital occupancy. (B) Modelled total community COVID-19 mortality compared to reported COVID-19 mortality. (C) Modelled COVID-19 mortality compared to reported COVID-19 mortality for residents of long-term care (LTC) facilities.

(A)

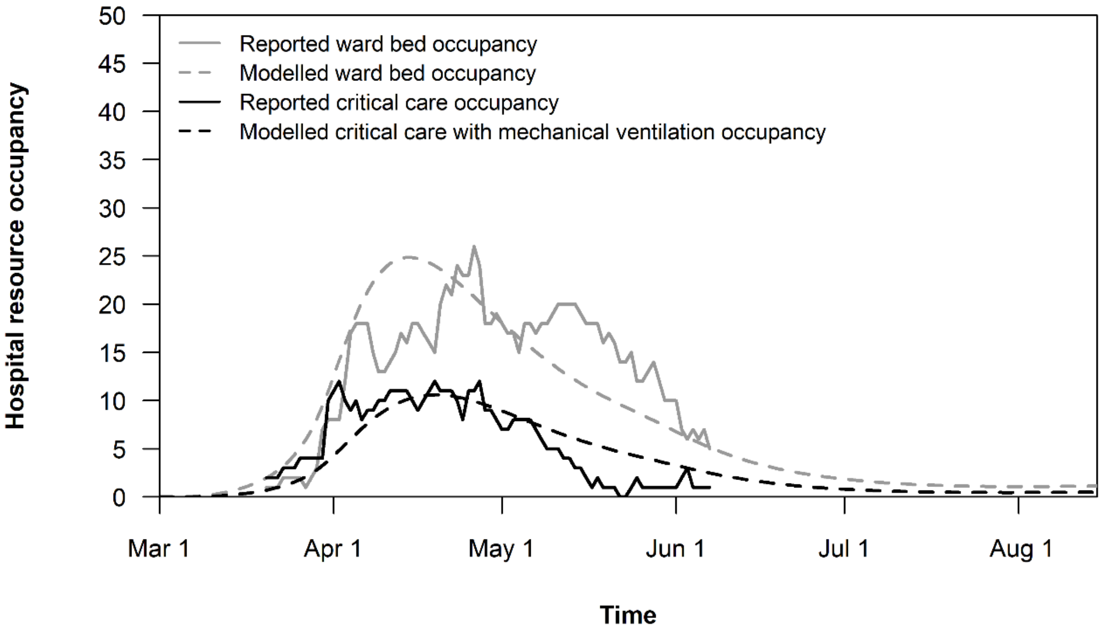

(B)

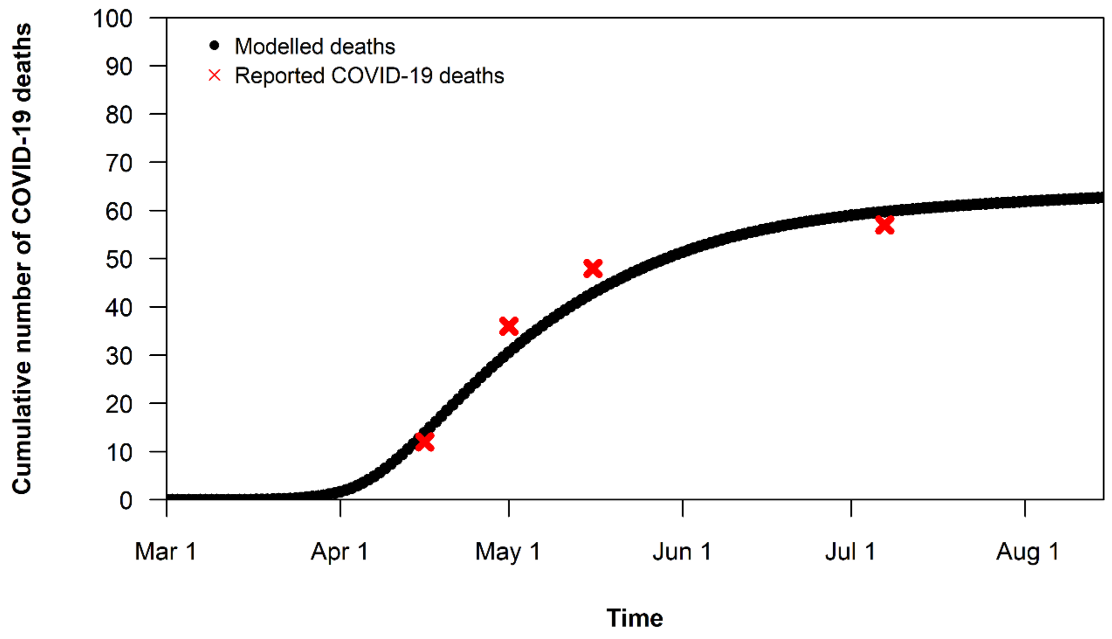
Modelled deaths
Reported COVID-19 deaths
Cumulative number of COVID-19 deaths
100
90
80
70
60
50
40
30
20
10
0
Mar 1
Apr 1
May 1
Jun 1
Jul 1
Aug 1
Time

(C)

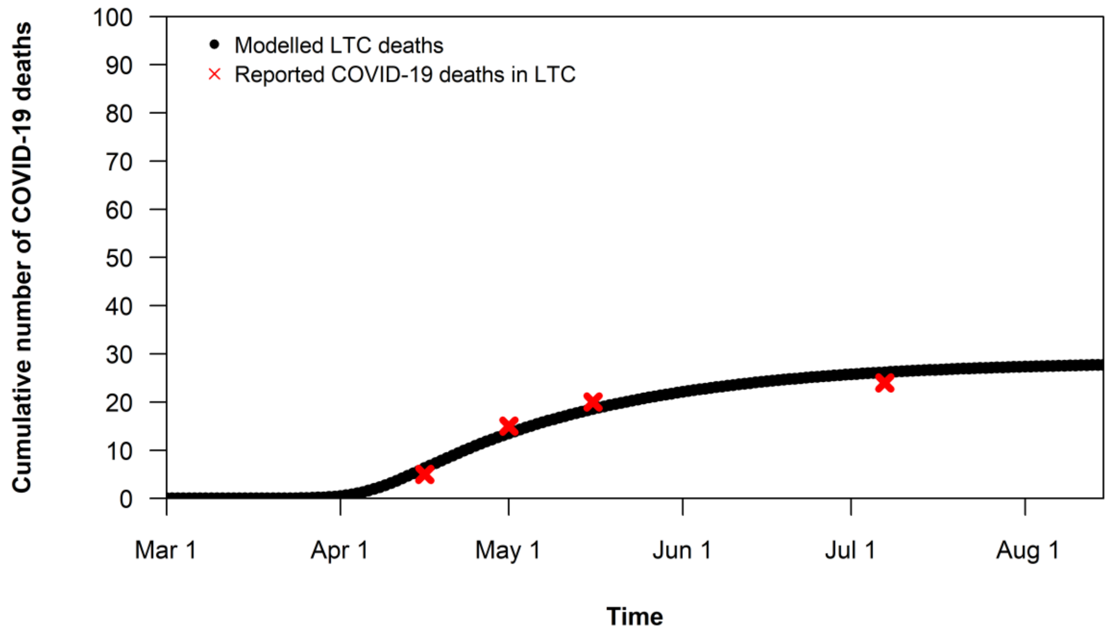
Modelled LTC deaths
Reported COVID-19 deaths in LTC
Cumulative number of COVID-19 deaths
100
90
80
70
60
50
40
30
20
10
0
Mar 1
Apr 1
May 1
Jun 1
Jul 1
Aug 1
Time

APPENDIX FIGURE 3. Participation in high-intensity physical distancing efforts in the calibration phase of the model-based analysis. All non-essential workplaces were ordered closed on March 23 [50]. (A) Proportion of the general population participating in high-intensity physical distancing, defined as having reduced their average number of daily contacts by 80% (from 12.6 to 2.5 contacts per day). Further, illustrated in panel (B), beginning May 16 [53], we assume that people engaged in high-intensity physical distancing wore a mask for 86% of their contacts and that people engaged in low-intensity physical distancing wore a mask for 38% of their contacts. (C) Proportion of long-term care residents engaged in high-intensity physical distancing. (D) Effective reduction in contacts from LTC resident engagement in physical distancing and the use of personal protective equipment.

(A)

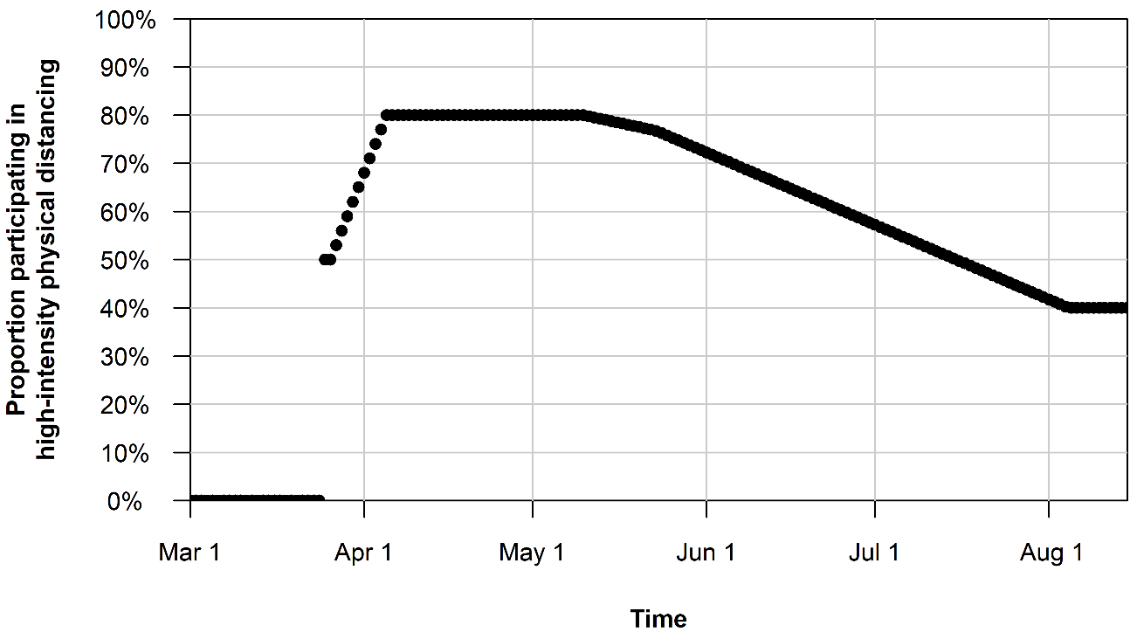

(B)

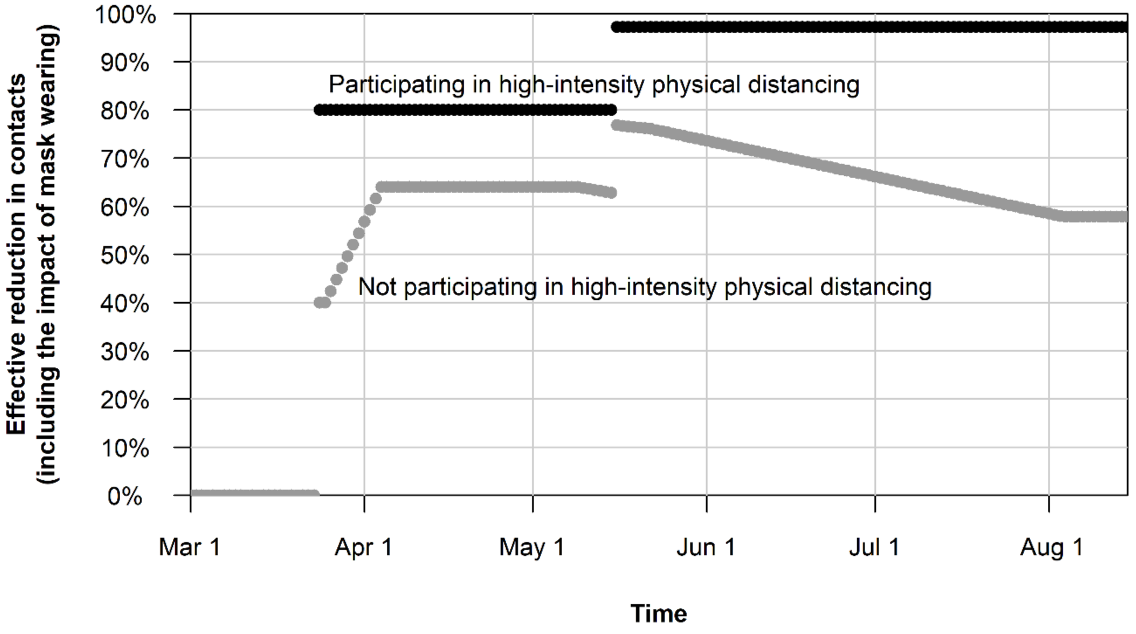

(C)

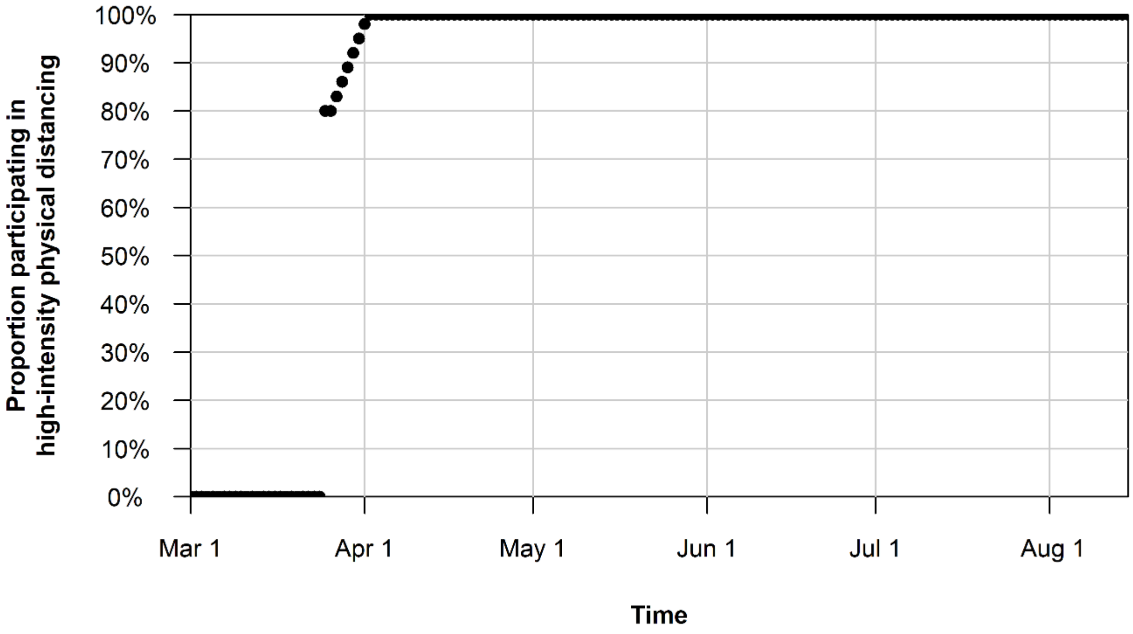
100%
90%
80%
70%
60%
50%
40%
30%
20%
10%
0%
Proportion participating in high-intensity physical distancing
Mar 1
Apr 1
May 1
Jun 1
Jul 1
Aug 1
Time

(D)

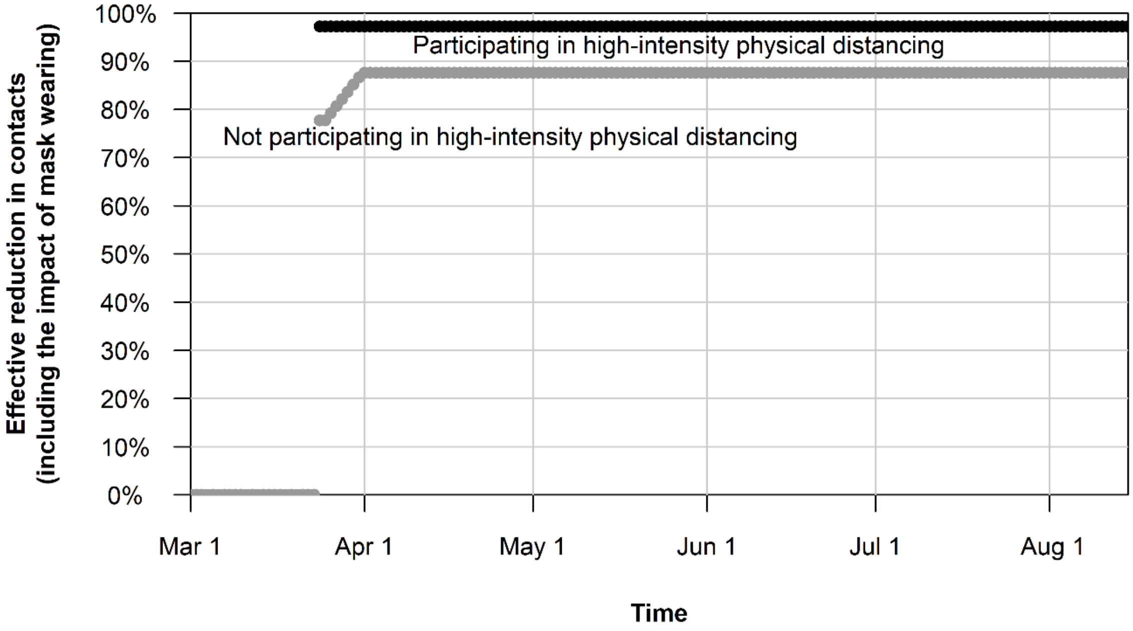
100%
90%
80%
70%
60%
50%
40%
30%
20%
10%
0%
Effective reduction in contacts (including the impact of mask wearing)
Participating in high-intensity physical distancing
Not participating in high-intensity physical distancing
Mar 1
Apr 1
May 1
Jun 1
Jul 1
Aug 1
Time

APPENDIX FIGURE 4. Google mobility report[48] for Middlesex County (which includes the City of London), Ontario Canada from March 1 to August 31 2020. All non-essential workplaces were ordered closed on March 23 [50]. **The baseline day is the day-of-week matched median value from the 5-week period Jan 3 – Feb 6, 2020.**

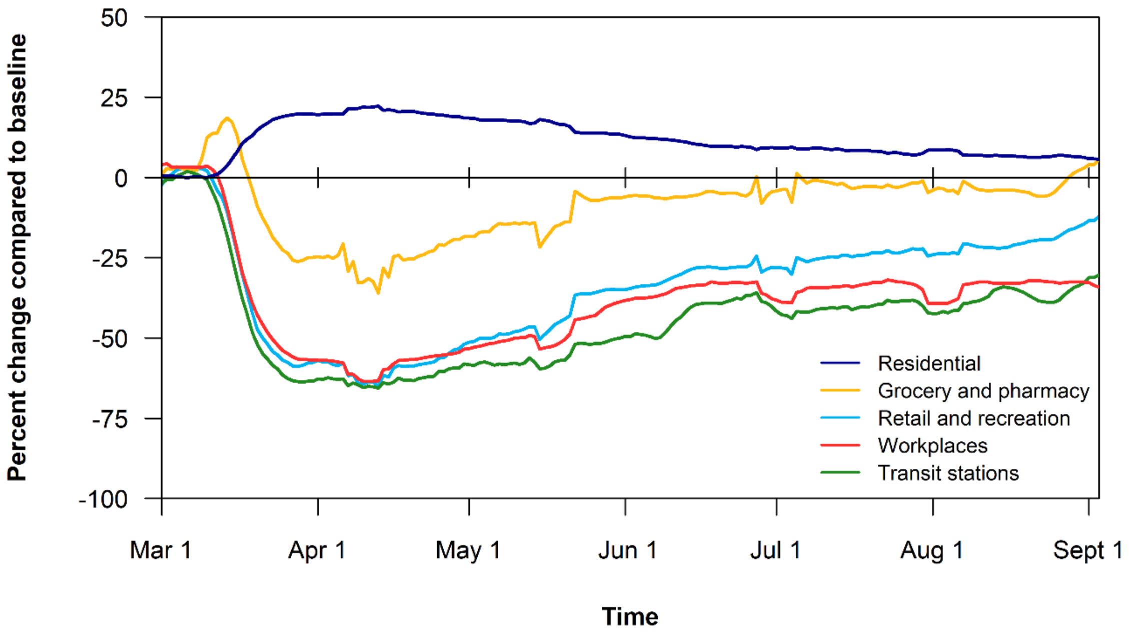

APPENDIX FIGURE 5. Number of new COVID-19 infections per day comparing different initial community conditions and physical distancing behaviours without the introduction of the university student population. (A) Varying the number of active cases in the community on August 15; and (B) Varying the proportion of the general population engaged in high-intensity physical distancing on August 15. High-intensity physical distancing is defined as reducing contacts by 75% (from 12.6 contacts per day to 3.15 contacts per day) and wearing a mask for 86% of remaining contacts.

(A)

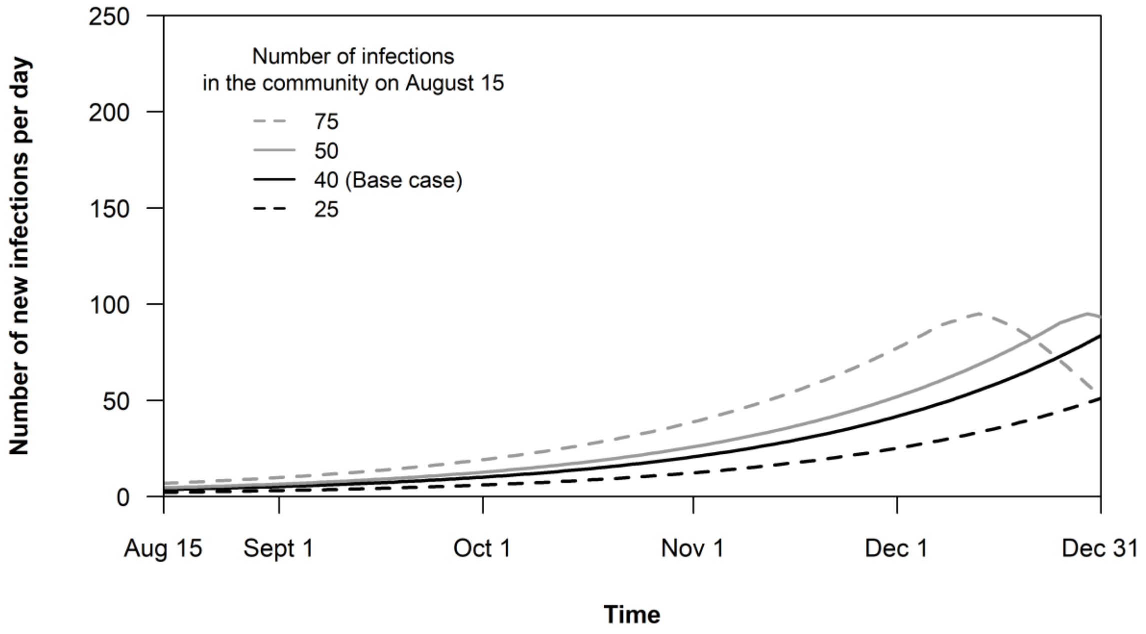

(B)

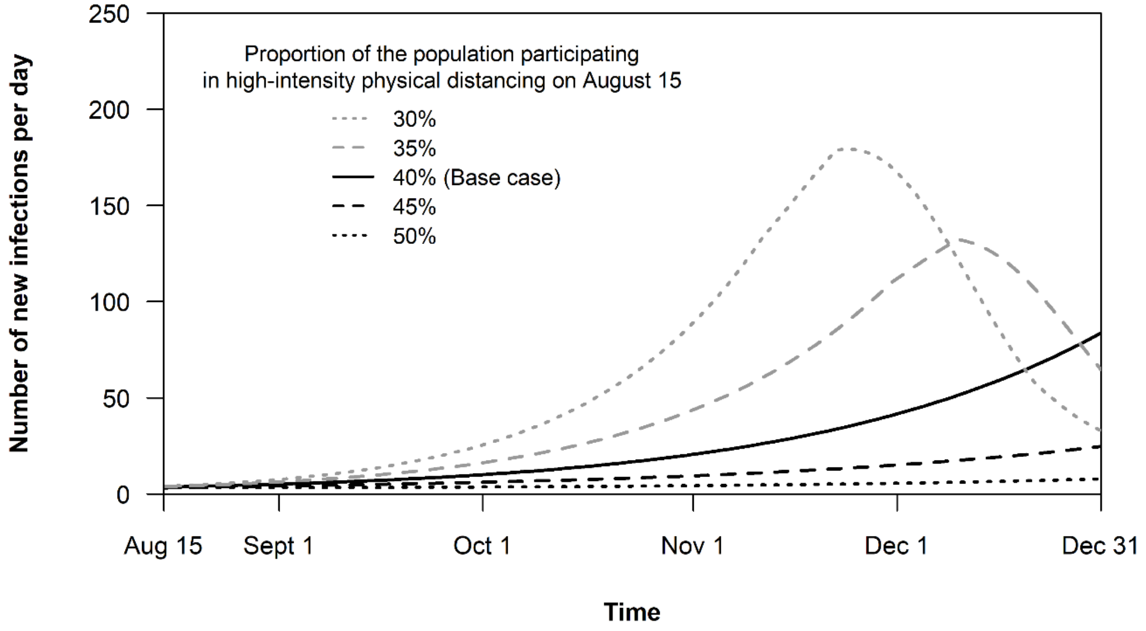

APPENDIX FIGURE 6. Number of COVID-19 patients medically indicated for critical care (mechanical ventilation or rental replacement therapy) each day comparing different initial community conditions and physical distancing behaviours without the introduction of the university student population. (A) Varying the number of active cases in the community on August 15; and (B) Varying the proportion of the general population engaged in high intensity physical distancing on August 15.

(A)

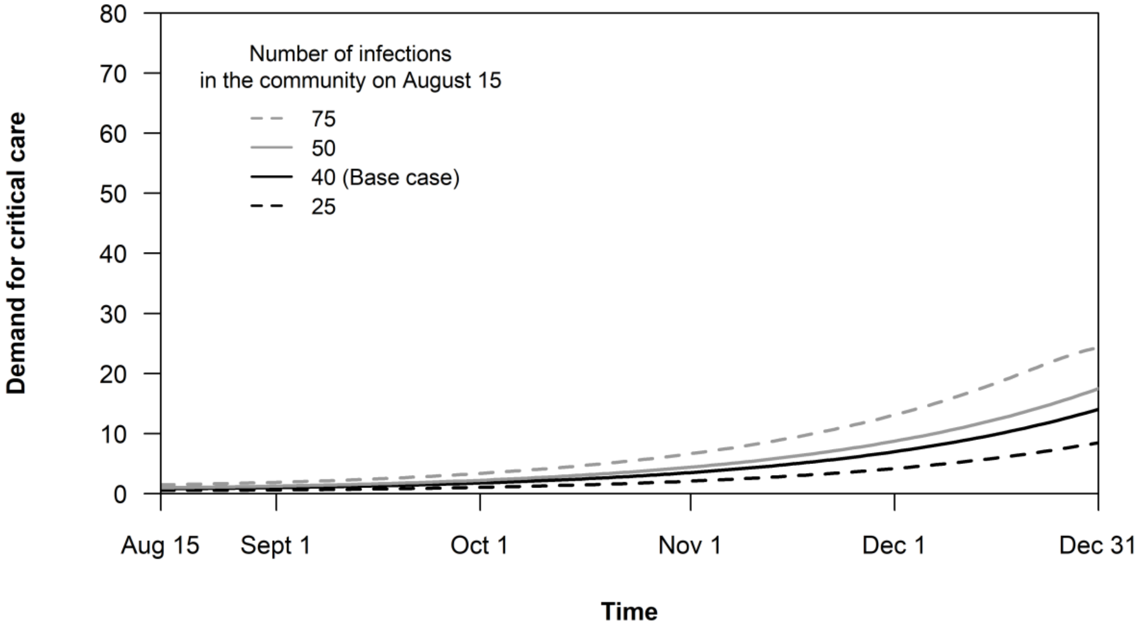

(B)

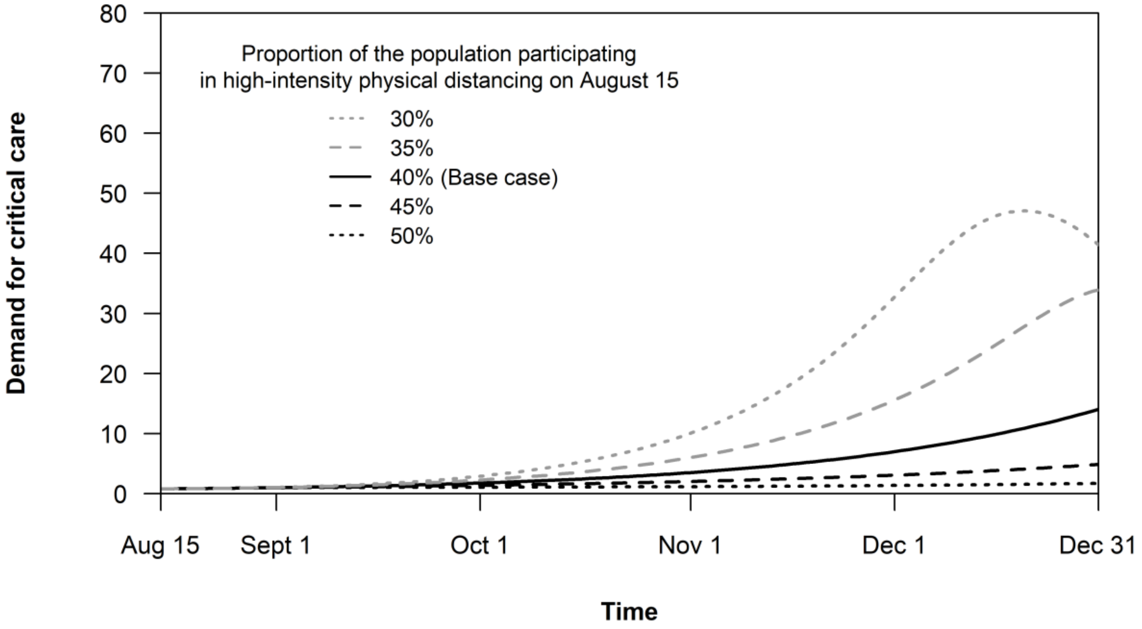

APPENDIX FIGURE 7. Cumulative number of COVID-19 deaths between August 15 and December 31 comparing different initial community conditions and physical distancing behaviours without the introduction of the university student population. (A) Varying the number of active cases in the community on August 15; and (B) Varying the proportion of the general population engaged in high intensity physical distancing on August 15.

(A)

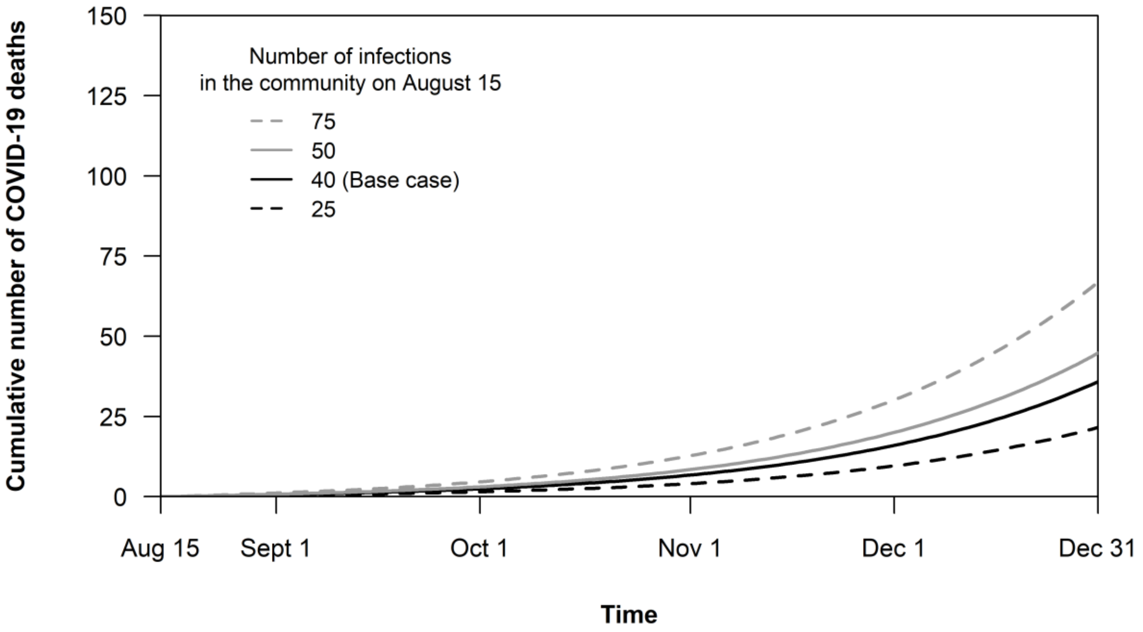

(B)

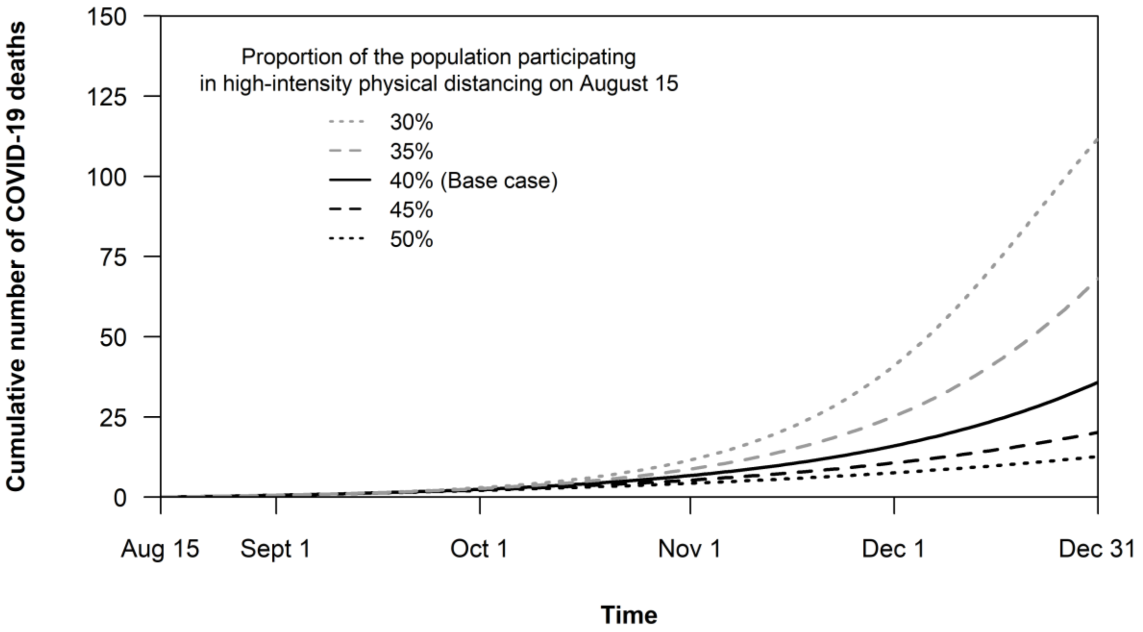

APPENDIX FIGURE 8. Cumulative COVID-19 deaths between August 15 and December 31 with and without the introduction of 20,000 university students on September 1. Only deaths occurring prior to December 31 are included in these figures. Scenarios presented along the X-axis of panel B correspond to scenarios presented in **Appendix Table 4**. The general population has the same features and COVID-19 prevention behaviours across all scenarios.

(A)

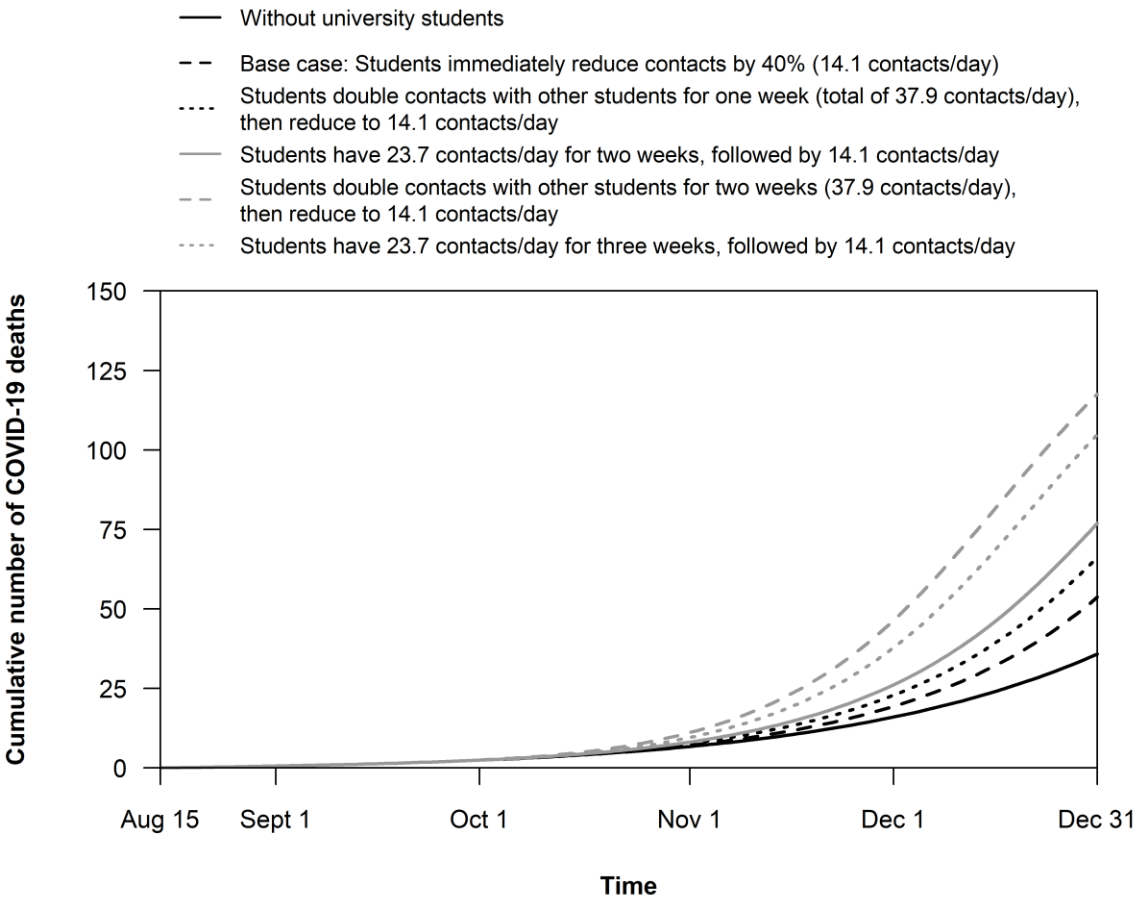

(B)

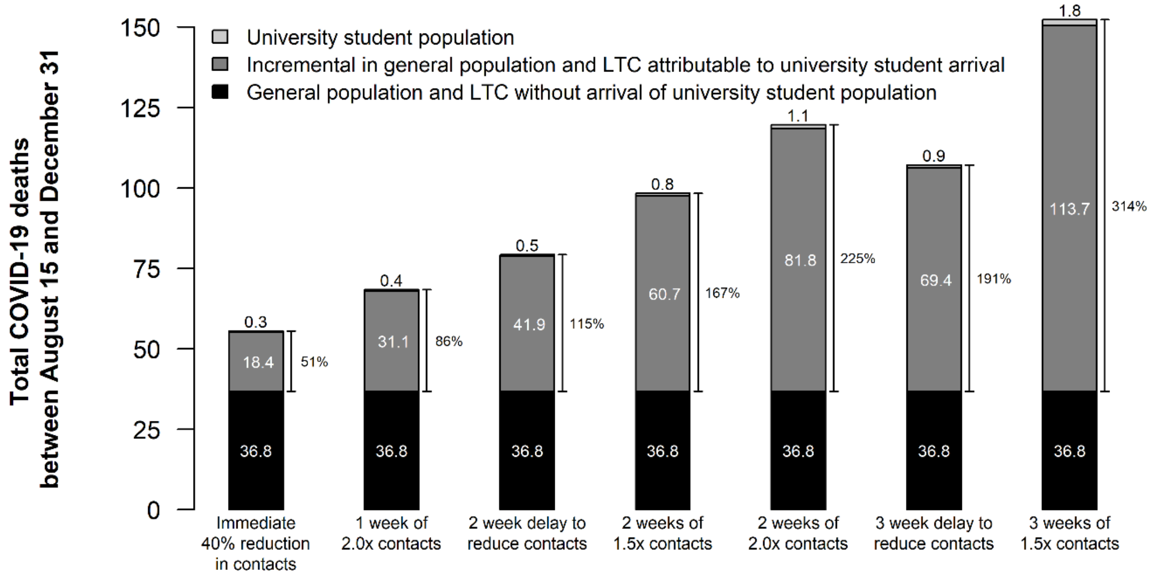

APPENDIX FIGURE 9. Number of new COVID-19 infections per day in the (A) general population and (B) university student population between August 15 and December 31 with and without the introduction of 20,000 university students on September 1. In the scenarios presented, students have twice the number of contacts with other students for two weeks (a total of 37.9 contacts per day) and then reduce to 14.1 contacts per day. Scenarios vary in the testing strategy employed by the university. The general population has the same features and COVID-19 prevention behaviours across all scenarios.

(A)

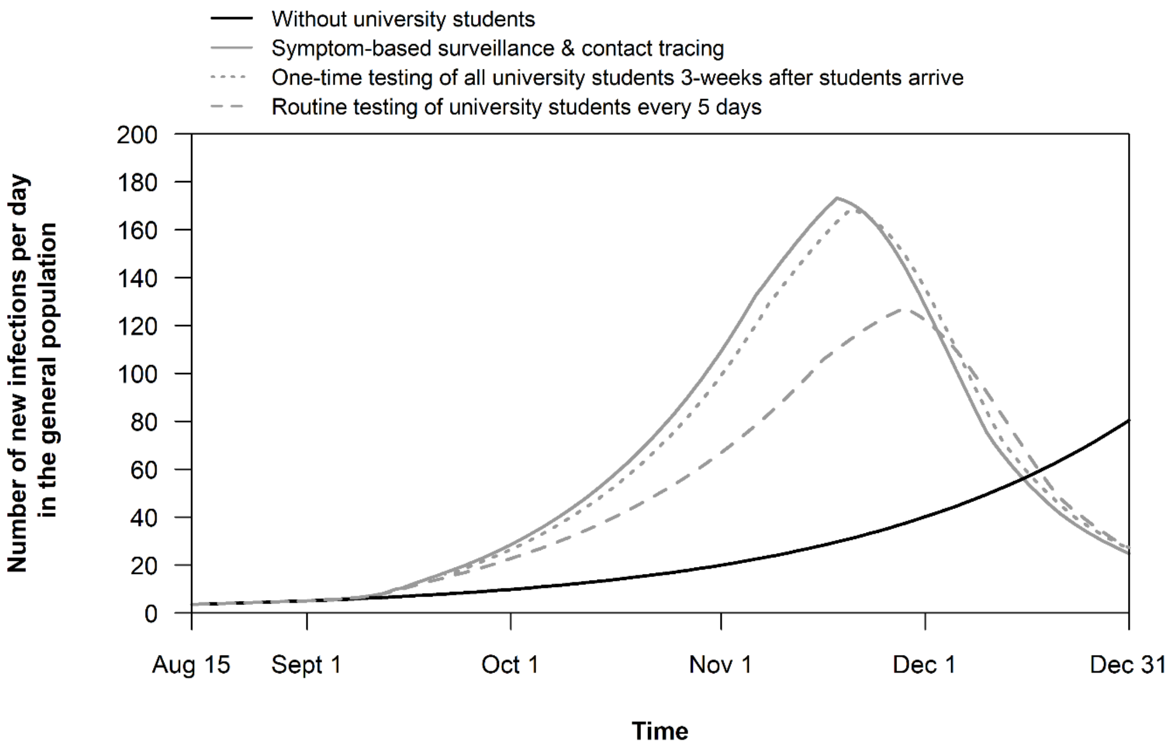

(B)

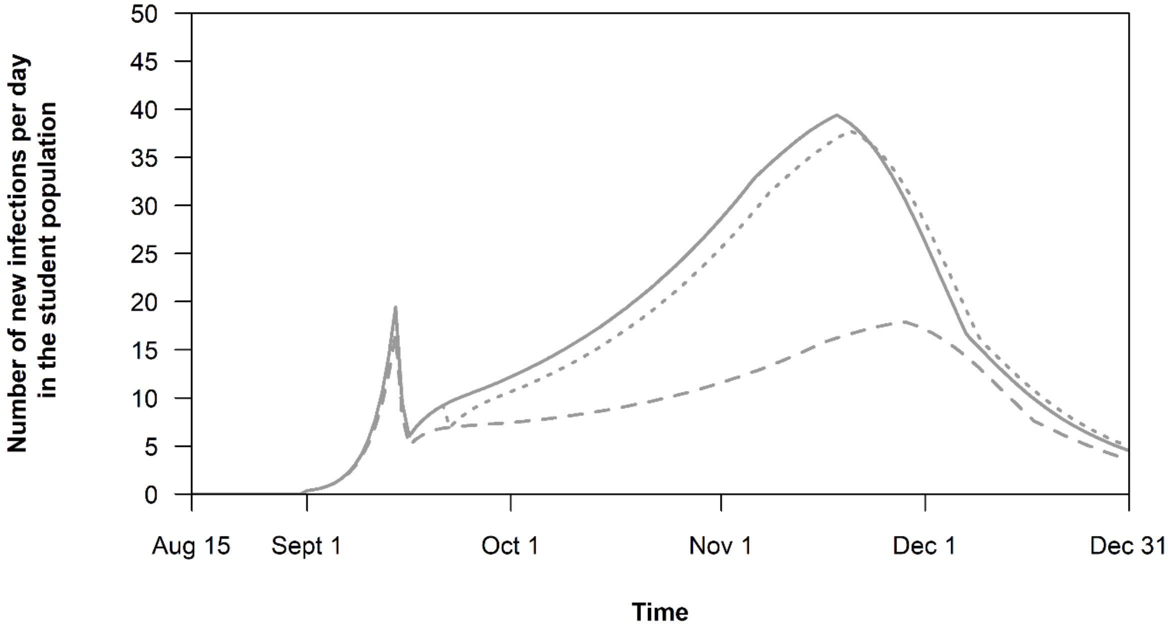

APPENDIX FIGURE 10. Cumulative number of COVID-19 infections (A) and deaths (B) between August 15 and December 31 with and without the introduction of 20,000 university students on September 1. Sensitivity analysis on university student behaviours in terms of contact reductions and mask wearing. Across all of these scenarios, the general population has base case behaviours. Numerical results are presented in **Appendix Table 6**.

(A)

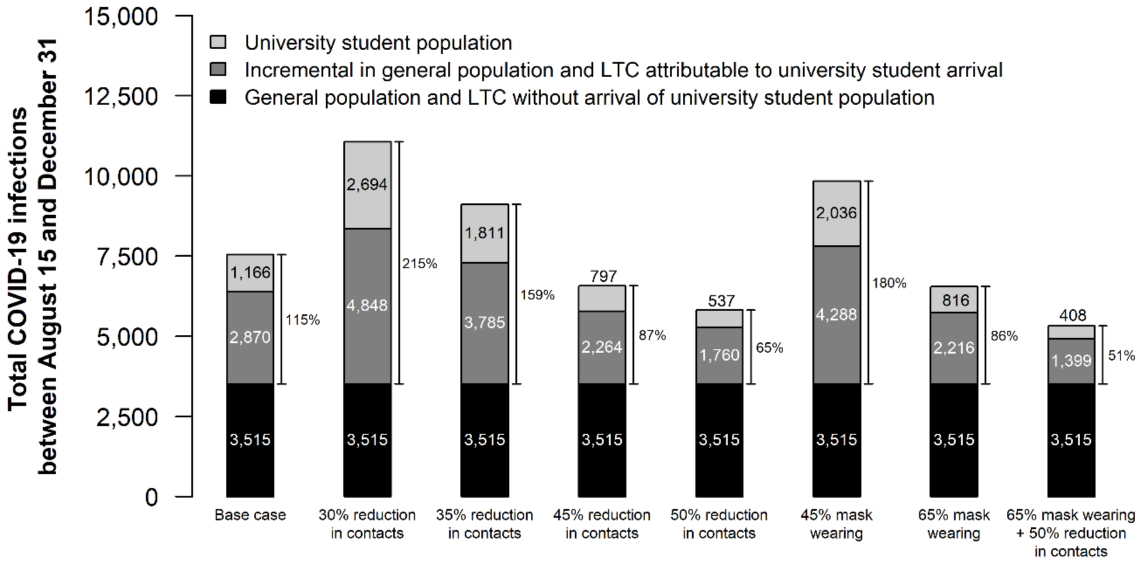

(B)

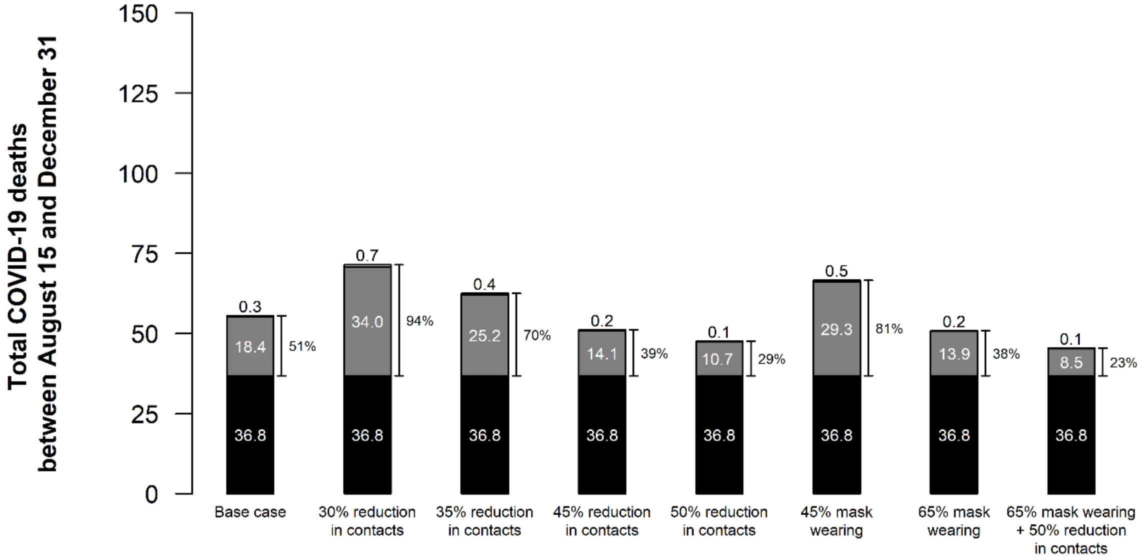

APPENDIX FIGURE 11. Cumulative number of COVID-19 infections (A) and deaths (B) between August 15 and December 31 with and without the introduction of 20,000 university students on September 1. Sensitivity analysis on general population behaviours and initial conditions. Across all of these scenarios, students have base case behaviours. Numerical results are presented in **Appendix Table 2**.

(A)

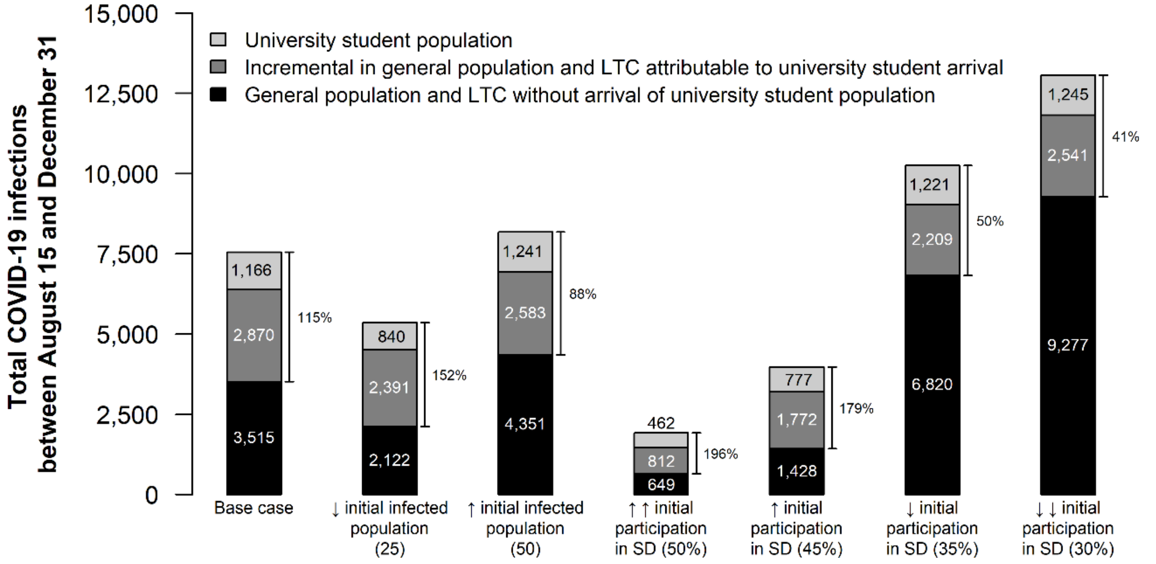

(B)

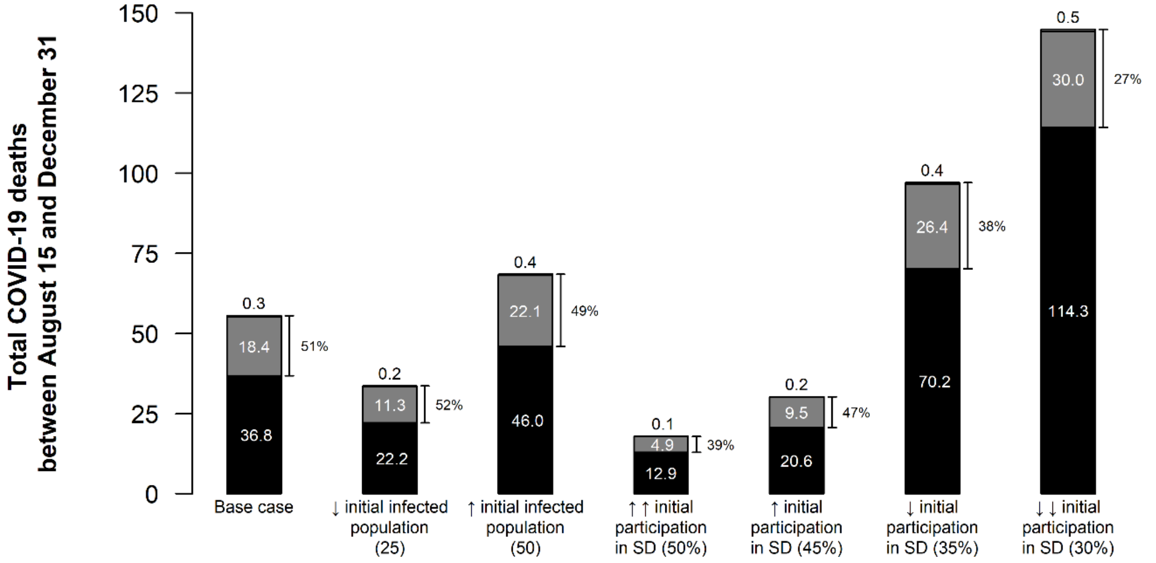

APPENDIX TABLE 1. Epidemic outcomes in a city of 500,000 in which 0.7% of the population live in long-term care (3500) comparing different initial community conditions and physical distancing (PD) behaviours without the university population over 4.5 months. Dates are presented in weeks from September 1 and four months is 17 weeks.

	**Base case**	**↓ initial infection population**	**↑ initial infection population**	**↑↑ initial participation in PD**	**↑ initial participation in PD**	**↓ initial participation in PD**	**↓↓ initial participation in PD**	**↓ threshold to increase PD effort**
**Scenario features**								
COVID-19 infections on August 15	40	25	50	40	40	40	40	40
Proportion of general population participating in high-intensity physical distancing on August 15	40%	40%	40%	50%	45%	35%	30%	40%
Trigger to increase general population participation in physical distancing		> 15 critical care occupancy → Increase physical distancing participation by 0.5% per day<br>> 10 deaths in past 10 days → Increase physical distancing participation by 1.0% per day						↓ threshold to 10 critical care occupancy
**Cumulative infections**								
General population	3,395	2,050	4,203	602	1,357	6,651	9,086	3,303
Long-term care	120	73	148	47	71	169	190	120
Total	3,515	2,122	4,351	649	1,428	6,820	9,277	3,423
**Daily infections**								
Peak number of infections	94	94	95	34	59	132	179	69
Date of peak infections (in weeks)	18.3	21.1	17.0	>22 weeks	>22 weeks	14.3	12.0	17.7
**Cumulative deaths**								
General population	24	14	30	7	12	50	87	24
Long-term care	13	8	16	6	9	20	28	13
Total	37	22	46	13	21	70	114	37
**Hospital occupancy**								
Peak number	37	37	37	16	25	51	68	29
Date of peak (in weeks)	21.3	>22 weeks	20.0	>22 weeks	>22 weeks	17.4	15.3	20.4
**Critical care (MV + RRT)**								
Peak number	25	24	25	9	16	35	47	19
Date of peak (in weeks)	21.7	>22 weeks	20.4	>22 weeks	>22 weeks	17.9	15.7	20.7
**Time to responsive behaviour triggers** (in weeks)								
> 10 COVID-19 deaths in past 10 days	18.6	21.4	17.3	Does not occur	Does not occur	14.6	12.0	18.6
> 15 COVID-19 patients in critical care	18.0	21.1	16.7	Does not occur	Does not occur	13.1	10.4	15.6

APPENDIX TABLE 2. Epidemic outcomes in a city of 500,000 in which 0.7% of the population live in long-term care (3500) with the introduction of 20,000 university students on September 1. Scenarios consider different initial community conditions and physical distancing (PD) behaviours in the general population. In all scenarios, the population of university students arriving on September 1 has the same features: university students arrive in the city with no COVID-19 infections, have 40% fewer contacts than the average number of normal contacts for a university student (average of 23.7 contacts reduced to 14.1 contacts), 58% of contacts are protected through mask wearing behaviour, and when COVID-19 begins to result in adverse outcomes in the community through critical care hospitalizations, university students increase their physical distancing efforts using the same triggers and at the same rate as the general population to a maximum reduction in contacts of 75% (average of 5.9 contacts). Dates are presented in weeks from September 1 and four months (to the end of the semester) is 17 weeks.

	**Base case**	**↓ initial infection population**	**↑ initial infection population**	**↑↑ initial participation in PD**	**↑ initial participation in PD**	**↓ initial participation in PD**	**↓↓ initial participation in PD**	**↓ threshold to increase PD effort**
**Scenario features**								
COVID-19 infections on August 15	40	25	50	40	40	40	40	40
Proportion of general population participating in physical distancing on August 15	40%	40%	40%	50%	45%	35%	30%	40%
Trigger to increase general population participation in physical distancing	> 15 critical care occupancy → Increase physical distancing participation by 0.5% per day<br>> 10 deaths in past 10 days → Increase physical distancing participation by 1.0% per day							↓ threshold to 10 critical care occupancy
**Cumulative infections**								
General population	6,231	4,408	6,765	1,399	3,101	8,847	11,608	5,366
Long-term care	153	106	169	62	100	182	209	149
University students	1,166	840	1,241	462	777	1,221	1,245	998
Total	7,551	5,354	8,175	1,923	3,978	10,251	13,063	6,513
**Daily infections**								
Peak number of infections	171	173	169	129	143	208	265	129
Date of peak infections (in weeks)	15.4	17.4	14.4	>22 weeks	18.7	13.1	11.6	15.4
**Cumulative deaths**								
General population	39	24	48	11	20	73	113	38
Long-term care	16	10	20	7	10	24	31	16
University students	0.3	0.2	0.4	0.1	0.2	0.4	0.5	0.3
Total	56	34	68	18	30	97	145	54
**Hospital occupancy**								
Peak number	57	58	56	41	49	71	92	47
Date of peak (in weeks)	18.6	20.7	17.7	>22 weeks	21.9	16.3	14.9	18.0
**Critical care (MV + RRT)**								
Peak number	38	39	38	26	31	48	63	30
Date of peak (in weeks)	19.1	21.1	18.1	>22 weeks	>22 weeks	16.9	15.3	18.4

**Time to responsive behaviour triggers** (in weeks)								
> 10 COVID-19 deaths in past 10 days	15.7	17.7	14.7	>22 weeks	19.0	13.1	11.3	15.7
> 15 COVID-19 patients in critical care	14.3	16.4	13.4	>22 weeks	18.0	11.6	9.7	12.6
				**Incremental compared to without the university students**				
**Cumulative infections**								
General population	2,836	2,358	2,562	797	1,744	2,195	2,522	2,063
Long-term care	33	33	21	15	28	14	19	29
University students	1,166	840	1,241	462	777	1,221	1,245	998
Total	4,036	3,232	3,824	1,274	2,550	3,430	3,786	3,090
**Daily infections**								
Peak number of infections	76	79	74	95	85	76	86	60
Date of peak infections (change in weeks)	-2.9	-3.7	-2.6	-9.7	-6.0	-1.1	-0.4	-2.3
**Cumulative deaths**								
General population	15	9	18	4	8	23	27	14
Long-term care	3.2	2.0	3.8	1.0	1.8	3.6	3.3	3.2
University students	0.3	0.2	0.4	0.1	0.2	0.4	0.5	0.3
Total	19	11	22	5	10	27	31	17
**Hospital occupancy**								
Peak number	20	21	19	24	24	21	24	17
Date of peak (change in weeks)	-2.7	-3.6	-2.3	-9.9	-6.3	-1.1	-0.4	-2.4
**Critical care (MV + RRT)**								
Peak number	13	14	13	17	15	14	16	10
Date of peak (change in weeks)	-2.6	-3.6	-2.3	-9.9	-6.3	-1.0	-0.4	-2.3
**Time to responsive behaviour triggers** (change in weeks)								
> 10 COVID-19 deaths in past 10 days	-2.9	-3.7	-2.6	Cannot be calculated	Cannot be calculated	-1.4	-0.7	-2.9
> 15 COVID-19 patients in critical care	-3.7	-4.7	-3.3	Cannot be calculated	Cannot be calculated	-1.6	-0.7	-3.0

APPENDIX TABLE 3. Epidemic outcomes between August 15 and December 31 in a city of 500,000 in which 0.7% of the population live in long-term care (3500) with the introduction of 20,000 university students on September 1. Scenarios vary on the number of COVID-19 infections in the student population when they arrive on September 1. Dates are presented in weeks from September 1 and four months (to the end of the semester) is 17 weeks.

		20,000 students arrive on September 1						
	**Without students**	**Base case**	**5 infected**	**10 infected**	**15 infected**	**20 infected**	**25 infected**	**30 infected**
**Scenario features**								
Number of infections in student population upon arrival on September 1		0	5	10	15	20	25	30
Immediate level of contact reduction among students		40% (from 23.7 → 14.1 contacts)	40%	40%	40%	40%	40%	40%
Contacts protected by mask wearing		57%	57%	57%	57%	57%	57%	57%
**Cumulative infections**								
General population	3,395	6,231	6,974	7,273	7,511	7,737	7,917	7,968
Long-term care	120	153	165	170	174	179	182	183
University students	0	1,166	1,376	1,491	1,589	1,681	1,759	1,809
Total	3,515	7,551	8,515	8,934	9,275	9,597	9,858	9,960
**Daily infections**								
Peak number of infections	94	171	177	178	180	184	188	189
Date of peak infections (in weeks)	18.3	15.4	14.4	13.6	13.0	12.6	12.1	11.7
**Cumulative deaths**								
General population	24	39	49	57	64	70	75	79
Long-term care	12.9	16.1	18.9	21.3	23.2	24.8	26.2	27.2
University students	0	0.3	0.4	0.5	0.6	0.7	0.8	0.8
Total	37	56	68	79	88	96	102	107
**Hospital occupancy**								
Peak number	37	57	59	59	59	61	62	62
Date of peak (in weeks)	21.3	18.6	17.6	16.9	16.1	15.7	15.3	14.9
**Critical care (MV + RRT)**								
Peak number	25	38	39	39	40	41	41	42
Date of peak (in weeks)	21.7	19.1	18.1	17.3	16.6	16.1	15.7	15.3
**Time to responsive behaviour triggers** (in weeks)								
> 10 COVID-19 deaths in past 10 days	18.6	15.7	14.7	13.9	13.3	12.9	12.4	12.0
> 15 COVID-19 patients in critical care	18.0	14.3	13.3	12.4	11.7	11.1	10.7	10.3

APPENDIX TABLE 4. Epidemic outcomes between August 15 and December 31 in a city of 500,000 in which 0.7% of the population live in long-term care (3500) with and without the introduction of 20,000 university students on September 1. Scenarios vary student participation in physical distancing immediately upon arrival to the community. In all scenarios, the general population begins with 40 COVID-19 infections on August 15, 40% of the general population are participating in high intensity physical distancing (75% reduction in daily contacts, from an average of 12.6 contacts to 3.8 contacts per day), 86% of contacts among high-intensity physical distancers and 38% of contacts among low -intensity physical distancers are protected through mask wearing behaviour, and when COVID-19 begins to result in adverse outcomes in the community through critical care hospitalizations, the fraction of the general population participating in high-intensity physical distancing and mask wearing behaviours increases to a maximum participation of 80%. Dates are presented in weeks from September 1 and four months (to the end of the semester) is 17 weeks.

		**20,000 students arrive on September 1**						
	**Without students**	**Immediate physical distancing (base case)**	**Students increase (2.0x) interactions for 1 week**	**Students delay physical distancing by 2 weeks**	**Students increase (1.5x) interactions for 2 weeks**	**Students increase (2.0x) interactions for 2 weeks**	**Students delay physical distancing by 3 weeks**	**Students increase (1.5x) interactions for 3 weeks**
**Scenario features**								
Number of infections in student population upon arrival on September 1		0	0	0	0	0	0	0
Reduction in overall student physical contacts of 24% (18.0 contacts) begins		Immediately	After 1 week	After 2 weeks	After 2 weeks	After 2 weeks	After 3 weeks	After 3 weeks
Burst of student-student contacts upon arrival		None	2.0x for 1 week	None	1.5x for 2 weeks	2.0x for 2 weeks	None	1.5x for 2 weeks
**Cumulative infections**								
General population	3,395	6,231	6,949	7,317	7,998	8,760	8,342	10,381
Long-term care	120	153	163	167	177	187	179	202
University students	0	1,166	1,386	1,545	1,843	2,221	2,050	3,138
Total	3,515	7,551	8,497	9,029	10,018	11,169	10,571	13,721
**Daily infections**								
Peak number of infections	94	171	176	180	194	216	206	275
Date of peak infections (in weeks)	18.3	15.4	14.4	13.6	12.4	11.1	11.9	9.6
**Cumulative deaths**								
General population	24	39	49	58	73	90	80	117
Long-term care	13	16	19	21	25	28	26	33
University students	0	0.3	0.4	0.5	0.8	1.1	0.9	1.8
Total	37	56	68	79	98	120	107	152
**Hospital occupancy**								
Peak number	37	57	58	59	63	70	67	86
Date of peak (in weeks)	21.3	18.6	17.6	16.7	15.6	14.3	15.0	12.7

**Critical care (MV + RRT)**								
Peak number	25	38	39	40	43	47	45	58
Date of peak (in weeks)	21.7	19.1	18.0	17.1	16.0	14.7	15.4	13.3
**Time to responsive behaviour triggers** (in weeks)								
> 10 COVID-19 deaths in past 10 days	18.6	15.7	14.7	13.9	12.7	11.4	12.1	9.9
> 15 COVID-19 patients in critical care	18.0	14.3	13.1	12.3	11.0	9.7	10.4	8.1

APPENDIX TABLE 5. Epidemic outcomes between August 15 and December 31 in a city of 500,000 in which 0.7% of the population live in long-term care (3500) with and without the introduction of 20,000 university students on September 1. Scenarios differ in the frequency with which students undergo routine testing for COVID-19. In the top third of the table, we assume students have an average 40% reduction in contacts compared to normal student physical interaction behaviour (average of 23.7 contacts reduced to 14.1 contacts per day) immediately upon arrival with no short-term increase in physical contacts upon arrival to the community. In the middle of the table, we assume that students double their contacts with other students for two weeks, and then implement a 40% reduction in their contacts. In the bottom section of the table, we assume that students double their contacts with other students for two weeks, and then implement a 40% reduction in their contacts and the general population is maintaining 50% participation in high-intensity physical distancing (compared to 40% in the base case). Dates are presented in weeks from September 1 and four months (to the end of the semester) is 17 weeks.

		20,000 students arrive on September 1						
	**Without students**	**Symptomatic surveillance & contact tracing**	**Test once 3-weeks after students arrive**	**Test every 28 days**	**Test every 14 days**	**Test every 7 days**	**Test every 5 days**	**Test every 3 days**
**Students immediately implement a 40% reduction in contacts compared to normal university student contact behaviour**								
**Cumulative infections**								
General population	3,395	6,231	6,154	5,913	5,635	5,297	5,094	4,790
Long-term care	120	153	152	151	148	146	144	141
University students	0	1,166	1,143	1,006	879	716	628	505
Total	3,515	7,551	7,449	7,069	6,663	6,159	5,866	5,436
**Daily infections**								
Peak number of infections	94	171	169	159	149	139	133	125
Date of peak infections (in weeks)	18.3	15.4	15.4	15.6	15.7	16.0	16.3	16.6
**Cumulative deaths**								
General population	24	39	38	37	36	34	33	31
Long-term care	13	16	16	16	16	15	15	15
University students	-	0.3	0.3	0.2	0.2	0.2	0.2	0.1
Total	37	56	55	53	52	49	48	46
**Hospital occupancy**								
Peak number	37	57	57	54	52	49	49	46
Date of peak (in weeks)	21.3	18.6	18.7	18.7	19.0	19.3	19.4	19.6
**Critical care (MV + RRT)**								
Peak number	25	38	38	36	34	32	31	30
Date of peak (in weeks)	21.7	19.1	19.1	19.3	19.3	19.6	19.7	20.0
**Time to responsive behaviour triggers** (in weeks)								
> 10 COVID-19 deaths in past 10 days	18.6	15.7	15.7	15.9	16.0	16.3	16.6	16.9
> 15 COVID-19 patients in critical care	18.0	14.3	14.4	14.6	14.7	15.1	15.3	15.7

	**Students double their contacts with other students for two weeks, then implement a 40% reduction in contacts compared to normal university student contact behaviour**							
**Cumulative infections**								
General population	3,395	8,760	8,570	8,253	7,745	7,156	6,918	6,428
Long-term care	120	187	185	183	178	174	174	170
University students	0	2,221	2,123	1,938	1,695	1,371	1,204	943
Total	3,515	11,169	10,878	10,375	9,618	8,700	8,296	7,541
**Daily infections**								
Peak number of infections	94	216	210	197	179	158	148	136
Date of peak infections (in weeks)	18.3	11.1	11.4	11.4	11.7	12.1	12.6	37.7
**Cumulative deaths**								
General population	24	90	86	84	78	70	65	57
Long-term care	13	28	28	28	26	25	24	23
University students	0	1.1	1.0	1.0	0.8	0.7	0.6	0.4
Total	37	120	115	112	105	96	91	81
**Hospital occupancy**								
Peak number	37	70	68	65	60	56	54	52
Date of peak (in weeks)	21.3	14.3	14.6	14.6	14.7	15.1	15.4	16.1
**Critical care (MV + RRT)**								
Peak number	25	47	46	44	40	37	35	33
Date of peak (in weeks)	21.7	14.7	15.0	15.0	15.1	15.6	15.9	16.4
**Time to responsive behaviour triggers** (in weeks)								
> 10 COVID-19 deaths in past 10 days	18.6	11.4	11.7	11.7	12.0	12.4	12.9	13.6
> 15 COVID-19 patients in critical care	18.0	9.7	10.0	10.0	10.1	10.7	11.1	11.9

	**Students double their contacts with other students for two weeks, then implement a 40% reduction in contacts compared to normal university student contact behaviour; the general population has a high level of participation in high-intensity physical distancing (50%)**							
**Cumulative infections**								
General population	602	5,464	5,243	4,885	4,362	3,345	2,782	2,061
Long-term care	47	148	145	143	138	120	108	92
University students	0	2,406	2,287	2,001	1,658	1,124	862	547
Total	649	8,018	7,675	7,029	6,157	4,589	3,751	2,700
**Daily infections**								
Peak number of infections	34	150	149	136	113	94	85	66
Date of peak infections (in weeks)	>22 weeks	14.9	15.4	15.7	16.6	18.3	19.6	21.9
**Cumulative deaths**								
General population	7	41	38	36	31	25	22	17
Long-term care	6	17	16	15	14	13	12	10
University students	0	0.9	0.8	0.7	0.6	0.4	0.3	0.2

Total	13	59	54	52	46	38	34	28
**Hospital occupancy**								
Peak number	16	56	56	51	42	33	30	25
Date of peak (in weeks)	>22 weeks	17.4	18.0	18.3	19.1	20.9	>22 weeks	>22 weeks
**Critical care (MV + RRT)**								
Peak number	9	36	36	33	26	21	19	16
Date of peak (in weeks)	>22 weeks	17.7	18.3	18.4	19.3	21.3	22.7	25.3
**Time to responsive behaviour triggers** (in weeks)								
> 10 COVID-19 deaths in past 10 days	Does not occur	15.1	15.7	16.0	16.9	18.6	19.9	22.1
> 15 COVID-19 patients in critical care	Does not occur	13.6	14.1	14.4	15.6	17.9	20.0	Does not occur

APPENDIX TABLE 6. Epidemic outcomes between August 15 and December 31 in a city of 500,000 in which 0.7% of the population live in long-term care (3500) with the introduction of 20,000 university students on September 1. Scenarios vary on student initial participation in physical distancing (PD) and the proportion of student contacts protected by mask wearing. Dates are presented in weeks from September 1 and four months (to the end of the semester) is 17 weeks.

		20,000 students arrive on September 1							
	**Without students**	**Base case student behaviours**	**↓↓ initial participation in PD**	**↓ initial participation in PD**	**↑ initial participation in PD**	**↑↑ initial participation in PD**	**↓ mask wearing**	**↑ mask wearing**	**↑ initial participation in PD & ↑ mask wearing**
**Scenario features**									
Number of infections in student population upon arrival on September 1		0	0	0	0	0	0	0	0
Immediate level of contact reduction among students		40% (from 23.7 → 14.1 contacts)	30% (23.7 → 16.6)	35% (23.7 → 15.4)	45% (23.7 → 13.0)	50% (23.7 → 11.9)	40%	40%	50% (23.7 → 11.9)
Contacts protected by mask wearing		57%	57%	57%	57%	57%	45%	65%	65%
**Cumulative infections**									
General population	3,395	6,231	8,198	7,141	5,630	5,130	7,641	5,583	4,773
Long-term care	120	153	164	158	149	144	161	148	140
University students	0	1,166	2,694	1,811	797	537	2,036	816	408
Total	3,515	7,551	11,057	9,110	6,576	5,811	9,838	6,547	5,322
**Daily infections**									
Peak number of infections	94	171	250	206	150	134	223	148	123
Date of peak infections (in weeks)	18.3	15.4	14.7	15.0	15.9	16.3	14.7	15.9	16.6
**Cumulative deaths**									
General population	24	39	53	45	35	33	48	35	31
Long-term care	12.9	16.1	18.2	17.1	15.5	14.9	17.6	15.4	14.5
University students	0	0.3	0.7	0.4	0.2	0.1	0.5	0.2	0.1
Total	37	56	71	63	51	48	67	51	45
**Hospital occupancy**									
Peak number	37	57	76	66	52	48	70	53	45
Date of peak (in weeks)	21.3	18.6	17.9	18.3	19.0	19.4	18.0	19.0	19.6
**Critical care (MV + RRT)**									
Peak number	25	38	51	44	35	32	47	34	30
Date of peak	21.7	19.1	18.3	18.7	19.4	19.9	18.6	19.4	20.0
**Time to responsive behaviour triggers** (in weeks)									
> 10 COVID-19 deaths in past 10 days	18.6	15.7	14.6	15.1	16.1	16.6	14.9	16.1	16.9
> 15 COVID-19 patients in critical care	18.0	14.3	13.1	13.7	14.9	15.4	13.4	14.9	15.7

TABLE 7. Economic value of COVID-19 infections averted through screening strategies of 5-day testing and one-time testing of students compared to a policy o f no routine asymptomatic testing (symptom-based surveillance and contact tracing only). Testing strategy is cost-effective if tests can be provided for less than the economic value per test. Scenarios vary the proportion of infections in the student population that are asymptomatic and timing and level of students contact reductions. We calculate the number of hospitalizations averted, critical care admissions averted, and COVID-19 deaths averted to be 3.75%, 1.7%, and 1.32% of general population infections averted, respectively, which includes hospitalizations and deaths which may occur after December 31 to all individuals infected prior to December 31. We calculated the economic costs of COVID-19 infections using an average direct cost of $10,411 per ward hospitalization, $74,157 per ICU hospitalization, and a cost of lost productivity of $2,090 per infection. We calculated the economic value of deaths averted using the observed age distribution of COVID-19 deaths at a value of $50,000 and $100,000 per life-year.

Scenario	Economic value (in $)			Economic value (in $) of COVID-19 deaths averted		Total economic value ($)		Economic value ($) per test	
	Infections averted	Hospital adm'n averted	Critical care adm'n averted	($50,000/ LY-gained)	($100,000/ LY-gained)	($50,000/ LY-gained)	($100,000/ LY-gained)	($50,000/ LY-gained)	($100,000/ LY-gained)
**5-day testing compared to no routine testing**									
**31% asymptomatic in students**									
Base case physical distancing (40% reduction in contacts immediately)	3,521,800	444,000	1,433,900	9,159,500	18,319,000	14,559,200	23,718,700	36	59
Two weeks of 2.0x activity among students, followed by 40% reduction in contacts	6,006,100	719,100	2,322,100	14,833,400	29,666,800	23,880,700	38,714,100	60	97
Low level of physical distancing (24% reduction in contacts immediately)	11,394,700	1,206,000	3,894,400	24,877,000	49,754,000	41,372,100	66,249,100	103	166
Two weeks of 2.0x activity among students, followed by 40% reduction in contacts; 50% of general population engaged in high-intensity physical distancing	8,918,800	1,047,200	3,381,700	21,602,200	43,204,300	34,949,900	56,552,000	87	141
**50% asymptomatic in students**									
Base case physical distancing (40% reduction in contacts immediately)	3,695,900	463,800	1,497,700	9,567,100	19,134,300	15,224,500	24,791,700	38	62
Two weeks of 2.0x activity among students, followed by 40% reduction in contacts	5,829,400	679,400	2,193,800	14,013,900	28,027,900	22,716,500	36,730,500	57	92
**80% asymptomatic in students**									
Base case physical distancing (40% reduction in contacts immediately)	4,042,800	505,400	1,632,100	10,425,500	20,851,000	16,605,800	27,031,300	42	68
Two weeks of 2.0x activity among students, followed by 40% reduction in contacts	6,769,500	803,400	2,594,200	16,571,200	33,142,400	26,738,300	43,309,500	67	108

	**One-time testing three weeks after student arrival compared to no routine testing**								
**31% asymptomatic in students**									
Base case physical distancing (40% reduction in contacts immediately)	212,700	30,200	97,400	622,300	1,244,600	962,600	1,584,900	48	79
Two weeks of 2.0x activity among students, followed by 40% reduction in contacts	606,900	74,400	240,100	1,533,700	3,067,400	2,455,100	3,988,800	123	199
Low level of physical distancing (24% reduction in contacts immediately)	387,800	45,400	146,600	936,600	1,873,100	1,516,400	2,452,900	76	123
Two weeks of 2.0x activity among students, followed by 40% reduction in contacts; 50% of general population engaged in high-intensity physical distancing	716,200	86,300	278,700	1,780,400	3,560,700	2,861,600	4,641,900	143	232
**50% asymptomatic in students**									
Base case physical distancing (40% reduction in contacts immediately)	106,100	13,700	44,100	281,900	563,900	445,800	727,800	22	36
Two weeks of 2.0x activity among students, followed by 40% reduction in contacts	688,300	85,400	275,700	1,761,200	3,522,300	2,810,600	4,571,700	141	229
**80% asymptomatic in students**									
Base case physical distancing (40% reduction in contacts immediately)	145,200	19,200	62,100	396,600	793,200	623,100	1,019,700	31	51
Two weeks of 2.0x activity among students, followed by 40% reduction in contacts	876,700	111,900	361,300	2,308,100	4,616,200	3,658,000	5,966,100	183	298

REFERENCES

1. Du Z, Wang L, Cauchemez S, Xu X, Wang X, Cowling BJ, et al. Risk for Transportation of 2019 Novel Coronavirus Disease from Wuhan to Other Cities in China. Emerg Infect Dis. 2020;26(5).
2. Tindale LC, Stockdale JE, Coombe M, Garlock ES, Lau WYV, Saraswat M, et al. Evidence for transmission of COVID-19 prior to symptom onset. Elife. 2020;9. doi: 10.7554/eLife.57149.
3. He X, Lau EHY, Wu P, Deng X, Wang J, Hao X, et al. Temporal dynamics in viral shedding and transmissibility of COVID-19. Nat Med. 2020;26:672-5.
4. Al-Jashi AA. COVID-19 in Ontario. Last updated September 10, 2020. [Accessed September 11, 2020] Availabile at: https://www.covid19inontario.com/home.
5. Western University. Facts & Figures 2019-2020. [Accessed September 11, 2020] Available at: https://www.uwo.ca/about/whoweare/facts.html.
6. Fanshawe College. About Fanshawe: London Campus. [Accessed September 11, 2020] Available at: https://www.fanshawec.ca/about-fanshawe/campus/london-campus.
7. Buitrago-Garcia D, Egli-Gany D, Counotte MJ, Hossmann S, Imeri H, Ipekci AM, et al. Occurrence and transmission potential of asymptomatic and presymptomatic SARS-CoV-2 infections: A living systematic review and meta-analysis. PLoS Med. 2020;17(9):e1003346.
8. Payne DC, Smith-Jeffcoat SE, Nowak G, Chukwuma U, Geibe JR, Hawkins RJ, et al. SARS-CoV-2 Infections and Serologic Responses from a Sample of U.S. Navy Service Members - USS Theodore Roosevelt, April 2020. MMWR Morb Mortal Wkly Rep. 2020;69(23):714-21.
9. Poletti P, Tirani M, Cereda D, Trentini F, Guzzetta G, Sabatino G, et al. Association of Age With Likelihood of Developing Symptoms and Critical Disease Among Close Contacts Exposed to Patients With Confirmed SARS-CoV-2 Infection in Italy. JAMA Netw Open. 2021;4(3):e211085.
10. Roxby AC, Greninger AL, Hatfield KM, Lynch JB, Dellit TH, James A, et al. Outbreak Investigation of COVID-19 Among Residents and Staff of an Independent and Assisted Living Community for Older Adults in Seattle, Washington. JAMA Intern Med. 2020;180(8):1101-5.
11. Kimball A, Hatfield KM, Arons M, James A, Taylor J, Spicer K, et al. Asymptomatic and Presymptomatic SARS-CoV-2 Infections in Residents of a Long-Term Care Skilled Nursing Facility - King County, Washington, March 2020. MMWR Morb Mortal Wkly Rep. 2020;69(13):377-81.
12. Intensive Care National Audit & Research Centre. ICNARC report on COVID-19 in critical care [July 17, 2020]. Available at: https://www.icnarc.org/Our-Audit/Audits/Cmp/Reports. 2020.
13. Statistics Canada. Detailed preliminary information on confirmed cases of COVID-19 (Revised), Public Health Agency of Canada. Table: 13-10-0781-01. [Accessed July 20, 2020]. Available at: https://www150.statcan.gc.ca/t1/tbl1/en/cv.action?pid=1310078101.
14. Docherty AB, Harrison EM, Green CA, Hardwick HE, Pius R, Norman L, et al. Features of 20 133 UK patients in hospital with covid-19 using the ISARIC WHO Clinical Characterisation Protocol: prospective observational cohort study. BMJ. 2020;369:m1985.
15. Cunningham JW, Vaduganathan M, Claggett BL, Jering KS, Bhatt AS, Rosenthal N, et al. Clinical Outcomes in Young US Adults Hospitalized With COVID-19. JAMA Internal Medicine. 2020;181(3):379-81.
16. Lauer SA, Grantz KH, Bi Q, Jones FK, Zheng Q, Meredith HR, et al. The Incubation Period of Coronavirus Disease 2019 (COVID-19) From Publicly Reported Confirmed Cases: Estimation and Application. Ann Intern Med. 2020;172:577-82.
17. Wolfel R, Corman VM, Guggemos W, Seilmaier M, Zange S, Muller MA, et al. Virological assessment of hospitalized patients with COVID-2019. Nature. 2020;581(7809):465-9.
18. To KK, Tsang OT, Leung WS, Tam AR, Wu TC, Lung DC, et al. Temporal profiles of viral load in posterior oropharyngeal saliva samples and serum antibody responses during infection by SARS-CoV-2: an observational cohort study. Lancet Infect Dis. 2020;20(5):565-74.

19. Wearing HJ, Rohani P, Keeling MJ. Appropriate models for the management of infectious diseases. PLoS Med. 2005;2(7):e174.
20. Keeling MJ, Rohani P. Modeling Infectious Diseases in Humans and Animals: Princeton University Press; 2008.
21. Novel coronavirus (SARS-CoV-2): Discharge criteria for confirmed COVID-19 cases. Technical report. . European Centre for Disease Prevention and Control, March 10, 2020. Available at: https://www.ecdc.europa.eu/en/publications-data/novel-coronavirus-sars-cov-2-discharge-criteria-confirmed-covid-19-cases. Report No.
22. Care in Canadian ICUs. Canadian Institute for Health Information (CIHI). 2016.
23. London Health Sciences Centre. Critical Care readiness: Expanding nursing staff to support COVID-19. [Accessed September 16, 2020]. Available at: https://www.lhsc.on.ca/news/critical-care-readiness-expanding-nursing-staff-to-support-covid-19.
24. Ventilator Stockpiling and Availability in theh US. Center for Health Security, Johns Hopkins Bloomberg School of Public Health, 2020 February 14, 2020. Report No.
25. Ng Y, Li Z, Chua YX, Chaw WL, Zhao Z, Er B, et al. Evaluation of the Effectiveness of Surveillance and Containment Measures for the First 100 Patients with COVID-19 in Singapore - January 2-February 29, 2020. MMWR Morb Mortal Wkly Rep. 2020;69(11):307-11.
26. Gaythorpe K, Imai N, Cuomo-Dannenburg G, Baguelin M, Bhatia S, Boonyasiri A, et al. Report 8: Symptom progression of COVID-19. Imperial College London COVID-19 Response Team. 2020.
27. Bi Q, Wu Y, Mei S, Ye C, Zou X, Zhang Z, et al. Epidemiology and transmission of COVID-19 in 391 cases and 1286 of their close contacts in Shenzhen, China: a retrospective cohort study. Lancet Infect Dis. 2020;20(8):911-9.
28. Herhalt C. Blood tests indicate one per cent of Ontario's population had COVID-19. CTV News. August 3, 2020. [Accessed September 11, 2020] Available at: https://toronto.ctvnews.ca/blood-tests-indicate-one-per-cent-of-ontario-s-population-had-covid-19-1.5049457.
29. Ontario Ministry of Health and Long-Term Care. COVID-19 Testing for Long-Term Care Home Staff. . July 16, 2020. [Accessed: August 26, 2020]. Available at: http://www.health.gov.on.ca/en/pro/programs/ltc/memo_20200716.aspx.
30. Centers for Disease Control and Prevention (CDC). Testing Residents: Interim SARS-CoV-2 Testing Guidelines for Nursing Home Residents and Healthcare Personnel [Accessed August 26, 2020] Available at: https://www.cdc.gov/coronavirus/2019-ncov/hcp/nursing-homes-testing.html.
31. Kucirka LM, Lauer SA, Laeyendecker O, Boon D, Lessler J. Variation in False-Negative Rate of Reverse Transcriptase Polymerase Chain Reaction-Based SARS-CoV-2 Tests by Time Since Exposure. Ann Intern Med. 2020;173(4):262-7.
32. Carver C, Jones N. Comparative accuracy of oropharyngeal and nasopharyngeal swabs for diagnosis of COVID-19. Centre for Evidence-Based Medicine. 2020;Available at: https://www.cebm.net/covid-19/comparative-accuracy-of-oropharyngeal-and-nasopharyngeal-swabs-for-diagnosis-of-covid-19/. Epub March 26, 2020.
33. Prem K, Cook AR, Jit M. Projecting social contact matrices in 152 countries using contact surveys and demographic data. PLoS Comput Biol. 2017;13(9):e1005697.
34. Edmunds WJ, O'Callaghan CJ, Nokes DJ. Who mixes with whom? A method to determine the contact patterns of adults that may lead to the spread of airborne infections. Proc Biol Sci. 1997;264(1384):949-57.
35. Beutels P, Shkedy Z, Aerts M, Van Damme P. Social mixing patterns for transmission models of close contact infections: exploring self-evaluation and diary-based data collection through a web-based interface. Epidemiol Infect. 2006;134(6):1158-66.
36. Edmunds WJ, Kafatos G, Wallinga J, Mossong JR. Mixing patterns and the spread of close-contact infectious diseases. Emerg Themes Epidemiol. 2006;3:10.

37. Mikolajczyk RT, Kretzschmar M. Collecting social contact data in the context of disease transmission: prospective and retrospective study designs. Social Networks. 2008;30(2):127-35.
38. Salathe M, Kazandjieva M, Lee JW, Levis P, Feldman MW, Jones JH. A high-resolution human contact network for infectious disease transmission. Proc Natl Acad Sci U S A. 2010;107(51):22020-5.
39. Aiello AE, Simanek AM, Eisenberg MC, Walsh AR, Davis B, Volz E, et al. Design and methods of a social network isolation study for reducing respiratory infection transmission: The eX-FLU cluster randomized trial. Epidemics. 2016;15:38-55.
40. Najafi M, Laskowski M, de Boer PT, Williams E, Chit A, Moghadas SM. The Effect of Individual Movements and Interventions on the Spread of Influenza in Long-Term Care Facilities. Med Decis Making. 2017;37(8):871-81.
41. Champredon D, Najafi M, Laskowski M, Chit A, Moghadas SM. Individual movements and contact patterns in a Canadian long-term care facility. AIMS Public Health. 2018;5(2):111-21.
42. Roberts AR, Ishler KJ, Adams KB. The Predictors of and Motivations for Increased Family Involvement in Nursing Homes. The Gerontologist. 2018;60(3):535-47.
43. Ontario Ministry of Health and Long-Term Care. COVID-19 case data. [Accessed August 26, 2020] Available at: https://covid-19.ontario.ca/data.
44. Public Health Ontario. Evolution of COVID-19 case growth in Ontario. Epidemiological summary. June 26, 2020. [Accessed: August 26, 2020]. Available at: https://www.publichealthontario.ca/en/data-and-analysis/infectious-disease/covid-19-data-surveillance.
45. Zhang J, Litvinova M, Liang Y, Wang Y, Wang W, Zhao S, et al. Changes in contact patterns shape the dynamics of the COVID-19 outbreak in China. Science. 2020;368(6498):1481-6.
46. Webster RK, Brooks SK, Smith LE, Woodland L, Wessely S, Rubin GJ. How to improve adherence with quarantine: rapid review of the evidence. Public Health. 2020;182:163-9.
47. Angus Reid Institute. COVID-19 Compliance: One-in-five Canadians making little to no effort to stop coronavirus spread. August 17, 2020. Available at: http://angusreid.org/covid-compliance/.
48. Mitze T, Kosfeld R, Rode J, Wälde K. Face Masks Considerably Reduce Covid-19 Cases in Germany. IZA Discussion Paper Series No 13319 Bonn, Germany: IZA Institute of Labour Economics 2020. doi: 10.1101/2020.06.21.20128181.
49. Goolsbee A, Syverson C. Fear, Lockdown, and Diversion: Comparing Drivers of Pandemic Economic Decline 2020. National Bureau of Economic Research Working Paper Series. 2020;No. 27432. doi: 10.3386/w27432.
50. Office of the Premier of Ontario. Press Release: Ontario Orders the Mandatory Closure of All Non-Essential Workplaces to Fight Spread of COVID-19. March 23, 2020. [Accessed September 11, 2020]. Available at: https://news.ontario.ca/en/release/56435/ontario-orders-the-mandatory-closure-of-all-non-essential-workplaces-to-fight-spread-of-covid-19.
51. Google. COVID-19 Community Mobility Reports. Middlesex County. [Accessed September 11, 2020]. Available at: https://www.google.com/covid19/mobility/.
52. Telus Insights. Data for Good: London (CSDUID 3539036 CSD Type: City). Reporting on data from February 22 to August 16, 2020.
53. Ontario Ministry of Health and Long-Term Care. Face coverings and face masks. [Published May 20, 2020; Last updated June 23, 2020; Accessed September 11, 2020]. Available at: https://www.ontario.ca/page/face-coverings-and-face-masks.
54. Middlesex-London Health Unit. Summary of COVID-19 cases in Middlesex-London. Available at: https://www.healthunit.com/covid-19-cases-middlesex-london.
55. Canadian Agency for Drugs and Technologies in Health. Guidelines for the Economic Evaluation of Health Technologies: Canada. 4th Edition. March 2017.

56. Aubin M, Cameron C, Coyle D, Dufort P, Husereau D, Jamieson P, et al. Working Group to Inform the Patented Medicine Prices Review Board (PMPRB) Steering Committee on Modernization of Price Review Process Guidelines. Final Report. March 2019.
57. Weinstein MC, Skinner JA. Comparative effectiveness and health care spending--implications for reform. N Engl J Med. 2010;362(5):460-5.
58. Neumann PJ, Sanders GD, Russell LB, Siegel JE, Ganiats TG. Cost-effectiveness in health and medicine: Oxford University Press; 2016.
59. Weekly Updates by Select Demographic and Geographic Characteristics. Provisional Death Counts for Coronavirus Disease 2019 (COVID-19) [Updated: December 30, 2020]. National Center for Health Statistics, Center for Disease Control and Prevention. [Accessed January 2, 2021] Available at: https://www.cdc.gov/nchs/nvss/vsrr/covid_weekly/index.htm.
60. Statistics Canada. Life tables, Canada, provinces and territories, 1980/1982 to 2016/2018. Catalogue no. 84-537-X. Available at: https://www150.statcan.gc.ca/n1/en/catalogue/84-537-X January 28, 2020.
61. Goldstein JR, Lee RD. Demographic perspectives on the mortality of COVID-19 and other epidemics. Proc Natl Acad Sci U S A. 2020;117(36):22035-41.
62. Gross domestic product price indexes, quarterly. Frequency: Quarterly. Table: 36-10-0106-01. [Accessed: January 2, 2021]. Available at: https://www150.statcan.gc.ca/t1/tbl1/en/tv.action?pid=3610010601
63. Implicit price indexes, gross domestic product, provincial and territorial. Frequency: Annual. Table: 36-10-0223-01. [Accessed: January 2, 2021]. Available at: https://www150.statcan.gc.ca/t1/tbl1/en/tv.action?pid=3610022301
64. Average weekly earnings by industry, annual. Frequency: Annual. Table: 14-10-0204-01. Geography: Canada, Province or territory. [Accessed January 2, 2021]. Available at: https://www150.statcan.gc.ca/t1/tbl1/en/tv.action?pid=1410020401.
